# Real-time and universal network for volumetric imaging from microscale to macroscale at high resolution

**DOI:** 10.1038/s41377-025-01842-w

**Published:** 2025-04-29

**Authors:** Bingzhi Lin, Feng Xing, Liwei Su, Kekuan Wang, Yulan Liu, Diming Zhang, Xusan Yang, Huijun Tan, Zhijing Zhu, Depeng Wang

**Affiliations:** 1 College of Energy and Power Engineering, Nanjing University of Aeronautics and Astronautics, Nanjing, China; 2 Key Laboratory of Novel Targets and Drug Study for Neural Repair of Zhejiang Province, School of Medicine, Hangzhou City University, Hangzhou, China; 3 Key Laboratory of Soybean Molecular Design Breeding, National Key Laboratory of Black Soils Conservation and Utilization, Northeast Institute of Geography and Agroecology, Chinese Academy of Sciences, Changchun, China; 4 Institute of Physics Chinese Academy of Sciences, Beijing, China

**Keywords:** Optical techniques, Microscopy

## Abstract

Light-field imaging has wide applications in various domains, including microscale life science imaging, mesoscale neuroimaging, and macroscale fluid dynamics imaging. The development of deep learning-based reconstruction methods has greatly facilitated high-resolution light-field image processing, however, current deep learning-based light-field reconstruction methods have predominantly concentrated on the microscale. Considering the multiscale imaging capacity of light-field technique, a network that can work over variant scales of light-field image reconstruction will significantly benefit the development of volumetric imaging. Unfortunately, to our knowledge, no one has reported a universal high-resolution light-field image reconstruction algorithm that is compatible with microscale, mesoscale, and macroscale. To fill this gap, we present a real-time and universal network (RTU-Net) to reconstruct high-resolution light-field images at any scale. RTU-Net, as the first network that works over multiscale light-field image reconstruction, employs an adaptive loss function based on generative adversarial theory and consequently exhibits strong generalization capability. We comprehensively assessed the performance of RTU-Net through the reconstruction of multiscale light-field images, including microscale tubulin and mitochondrion dataset, mesoscale synthetic mouse neuro dataset, and macroscale light-field particle imaging velocimetry dataset. The results indicated that RTU-Net has achieved real-time and high-resolution light-field image reconstruction for volume sizes ranging from 300 μm × 300 μm × 12 μm to 25 mm × 25 mm × 25 mm, and demonstrated higher resolution when compared with recently reported light-field reconstruction networks. The high-resolution, strong robustness, high efficiency, and especially the general applicability of RTU-Net will significantly deepen our insight into high-resolution and volumetric imaging.

## Introduction

Owing to the snapshot volumetric acquisition, light-field imaging technique has successfully advanced the development of three-dimensional measurement, ranging from microscale^1^ to macroscale light-field imaging^2^. These light-field imaging systems have shown the ability to capture detailed and comprehensive images of structures and functions^3^, including neuroimaging in the biological domain^4^ and flow dynamics imaging in the mechanical domain^5^. In particular, high-resolution volumetric imaging of fluorescent protein-labeled neurons of zebrafish larvae and worms has been demonstrated by light-field microscopy^3^. In addition, the combination of light-field camera and particle imaging velocimetry (PIV) technique has enabled flow speed calculation for complex flows in three dimensions, such as vortex ring^6^, and shock-boundary interaction flow^7^. Although light-field imaging systems have been effective in these applications, the limited spatial resolution hinders its widespread adoption at the desired level of accuracy.

Multiple methods have been proposed to improve the spatial resolution of light-field modality through hardware system innovation and image reconstruction software optimization. System innovation approaches can enhance resolution^8^, but the increased complexity and cost unavoidably hinder the application of light-field technology. Current light-field image reconstruction methods mainly rely on convolutional neural network (CNN) based deep learning approaches, such as VCD-Net^3^, HyLFM-Net^9^, and VsLFM^10^. The results obtained by these networks tested on various currently available public datasets (Table S1) leave much to be desired (Table S2). VCD-Net can realize high-speed (200 Hz) 3D imaging of neuronal activity, cardiac blood flow, and other rapid biological processes, but it employed a typical U-Net structure^11^ after the data was pre-upsampled through stacked Conv2D and PixelShuffle^12^, which can induce noise amplification and additional cost during computation. HyLFM-Net utilized ConvTranspose rather than PixelShuffle during upscale sampling, which impaired resolution by partially cutting off high-frequency signals. Instead of using pre-upsampling, HyLFM-Net used progressive upsampling, making the network more complex for training. The 3D residual blocks in HyLFM-Net consisted of Conv3D, which can reduce the computation efficiency when compared to the conv2D used in VCD-Net. HyLFM has demonstrated high-quality 3D reconstruction through imaging of zebrafish neural activity and cardiac dynamics, achieving volume imaging rates up to 100 Hz. However, HyLFM-Net employed plenty of 3D convolution operations in the network, which can lead to an increase in the number of network parameters and training time. VsLFM took a new route to improve the spatial resolution of light-field images. Instead of working on the reconstruction procedure, VsLFM focused on the processing of raw light-field images and generated multiple-view data through virtual scanning. Thus, VsLFM solely improved the resolution of the raw light-field image, therefore, its 3D volume reconstruction still involved the time-consuming iteration procedure, whose efficiency was thousands of times lower than that of VCD-Net and HyLFM-Net. VsLFM has achieved near-diffraction-limited high-resolution with strong robustness to optical distortion and sample motion, which has been demonstrated in the imaging of zebrafish hearts, Drosophila brains, and mouse livers. Although VsLFM has made important progress in improving the resolution and applicability of LFM, its training highly relies on high-quality data from scanning light-field microscope, which was not widely available.

Although all these reported methods improved the spatial resolution of light-field images, they have two common drawbacks. First, all these methods only demonstrated their feasibility in microscale image reconstruction and their performance on large scale remained unknown. The limited application of these networks severely prevented light-field imaging from benefiting more research domains and prohibited the development of interdisciplinary research. For instance, light-field PIV is a commonly used technique for flow dynamic measurements, such as microfluidics experiments in the biological domain and macroscale water tunnel experiments in the mechanically related domain. When the same network being applicable to both scales of light-field image reconstruction, a fair comparison of flow phenomena in both the microfluidics experiment and the water tunnel experiment can be established. This would result in a collaboration between the two domains to explore the flow mechanism at different scales. Second, all these reported methods employed CNN-based networks that relied on traditional loss functions for network training. Typically, the design of traditional linear loss functions requires a specific optimization target defined for CNN to minimize the difference between predicted and ground truth images, which involves averaging all plausible outputs and thus produces blurring results^13^. Specifically, when minimizing the Euclidean distance to its nearest neighbor, traditional loss function would make the optimization stay or vibrate at the average of nearby source samples, thereby leading to blurry patches^14,15^. Additionally, because traditional linear loss functions do not allow learn-to-adapt, determining a proper linear loss function was a low-efficiency and time-consuming procedure, and a lot of manual effort was required to fine-tune the loss function, making the whole training process low efficiency^16^. Therefore, the optimization of CNN-based networks was a lengthy procedure. In contrast, generative adversarial networks (GANs) offer an adaptive nonlinear loss function, which allows adaptation and tunability according to the data during the training procedure^17^. This enabled GANs to be used in plenty of tasks that would require different kinds of loss functions in conventional methods^16,18^, thereby significantly reducing the time needed for loss function determination. By cooperating with a generative model, GAN can be readily customized for applications that otherwise require different types of loss functions. Though GAN has been widely utilized in various fields, its potential in high-resolution light-field image reconstruction remains unexplored.

Here, we introduce a novel approach called Real-Time and Universal network (RTU-Net), which can reconstruct 3D high-fidelity content from 2D light-field raw data at uniform spatial resolution improvement over a broad range of applications across different dimensions, ranging from microscale to macroscale. Currently, RTU-Net is the only network that can generalize the weights for multiscale light-field reconstruction and has revealed strong robustness when the network was trained on data at a fixed scale but tested on data at different scales or from variant imaging sources. Specifically, we tested the performance of RTU-Net with microscale datasets including tubulin and mitochondrion, mesoscale synthetic mouse neural imaging data, and macroscale light-field PIV data. Results proved that RTU-Net has enabled faster and higher resolution light-field image reconstruction when compared to existing end-to-end networks (Table S3). Additionally, it eliminated the time-consuming iterations involved in the reconstruction process when compared to physics-based approaches. Due to its multiscale reconstruction capability and high accuracy, RTU-Net will significantly enhance the advancement of volumetric image reconstruction.

## Results

### Principle of RTU-Net

**Fig. 1:**
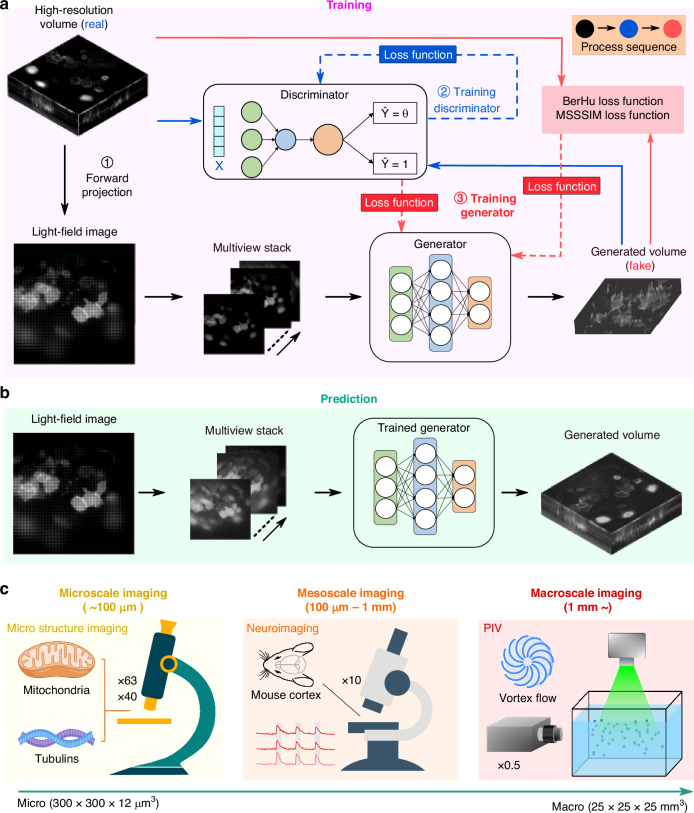
Schematic drawing shows the layout of RTU-Net. **a** Simplified schematic of RTU-Net training procedure. The high-resolution 3D stationary samples can be trained using synthetic or experimental methods. The network is optimized with three loss functions. **b** The schematic shows the prediction procedure of RTU-Net. **c** Examples highlights the working scale of RTU-Net, including structural imaging of mitochondria and microtubule proteins in microscale (left), neuroimaging of the rat brain in mesoscale (center), and PIV measurement for particle imaging in macroscale (right)

RTU-Net takes raw light-field image as the input and outputs reconstructed high-resolution volumetric image. RTU-Net is trained using a GAN, which has one generator and one discriminator, both of which are CNN networks and are trained and optimized through loss function iteratively. Before training, we create the training dataset by converting the high-resolution 3D images (obtained synthetically or experimentally) of stationary samples into 2D light-field images using the wave optics model of LFM (Fig. 1, “Methods” and Fig. S1)^17^. The light-field image is subsequently organized into sub-images of varying views, which are then fed into the generator. The generator initially extracts the characteristics of the sub-images and subsequently improves the resolution through a combination of convolution and up-sampling strategies (Figs. S2 and S3). Following that, spatial information obtained from multiple angles is combined using cascaded convolutional layers to produce 3D image stacks with varying depths, resulting in the generated volume (fake). The RTU-Net is trained using three strategies of optimization. One optimization method involves training the discriminator by alternating between using genuine and fake data as input. The goal is to enhance the discriminator’s ability to accurately distinguish between fake and real data. When real (fake) data is provided as input, the discriminator is optimized to accurately identify the input as 1 (0). The second optimization process enhances the performance of the generator by utilizing BerHu and multiscale structural similarity index measure (MSSSIM) loss functions. This process utilizes the ground truth (real) and the output volume (fake) as the basis for optimization, aiming to push the generator to produce a volume that closely resembles the ground truth volume. The last optimization improves the generator by using the discriminator as the basis for optimization. When the artificially generated volume produced by the generator is fed into the discriminator, the discriminator will output a value ranging from 0 to 1. This value is then passed back to the generator, which uses it to improve itself by minimizing the binary cross-entropy (BCE) loss function to generate a volume that matches the ground truth. Through iterative minimization of these loss functions, the network is progressively tuned until the discriminator is unable to distinguish between the ground truth and the artificially generated volume provided by the generator. At this point, the training process comes to a halt. Finally, the network that has undergone training is employed to perform predictions (Fig. 1b). We have demonstrated the feasibility of RTU-Net in the reconstruction of high-resolution light-field images in multiple applications over different scales, including microscale (~ 300 µm) structural and functional data, mesoscale neuroimaging data (~ 1 mm), and macroscale (~ 3 cm) PIV dataset.

### RTU-Net demonstrated real-time superior resolution in reconstructing microscale tubulin with different densities and diameters

**Fig. 2:**
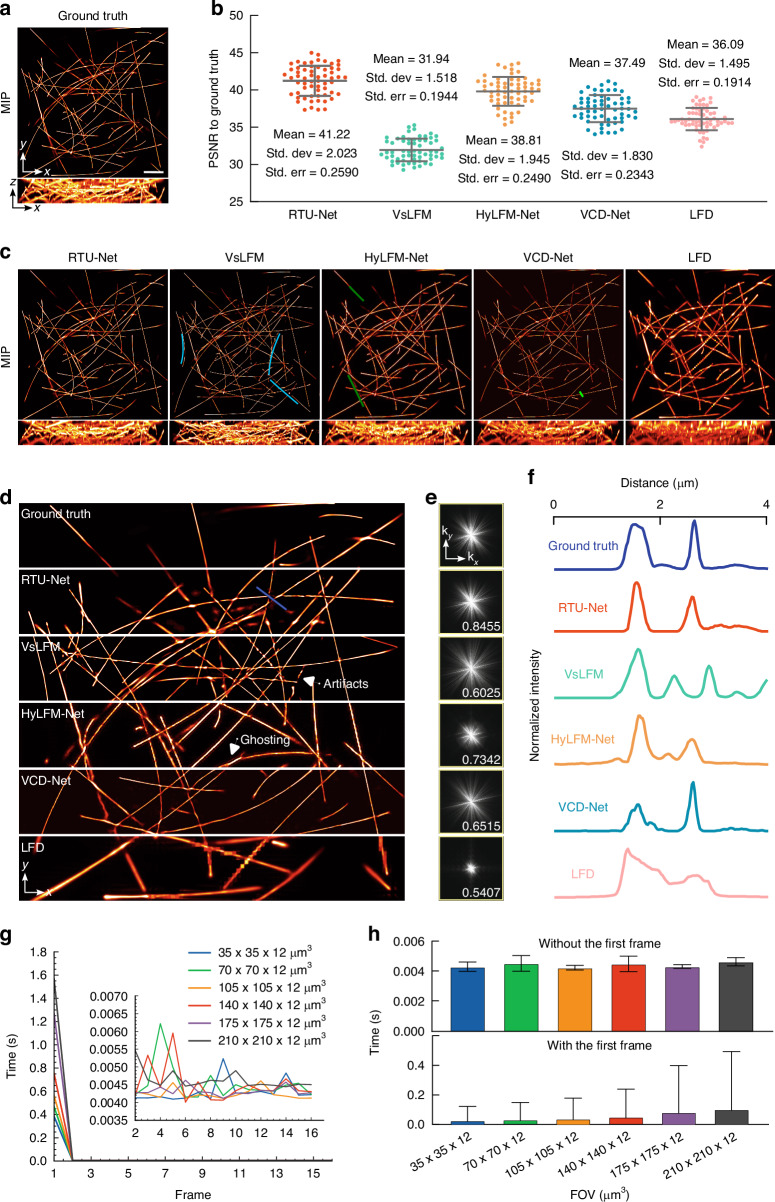
The performance of RTU-Net on synthetic tubulins. **a** Orthogonal MIPs of 1-μm-diameter synthetic tubulins, acquired by sLFM with a ×63/1.4 NA oil-immersion objective in ideal imaging conditions. **b** PSNR of results obtained by RTU-Net, VsLFM, HyLFM-Net, VCD-Net, and LFD. The center line represents the median, the box limits represent the lower and upper quartiles, and the whiskers represent 1.5-fold the interquartile range, *n* = 61. **c** MIPs obtained by RTU- Net VsLFM, HyLFM-Net, VCD-Net, and LFD trained on the same type of sample. Blue and green lines label the defects of the reconstructed images. **d** The enlarged view of images for ground truth, RTU-Net, VsLFM, HyLFM-Net, VCD-Net, and LFD. **e** The Fourier transforms of the whole FOV image for different methods. The SSIM values between the FFT of the ground truth image and the FFT of each method are displayed. **f** The normalized intensity profile of the blue line labeled feature in (**d**). **g** The curves of reconstruction time versus frame levels applied for different FOV (inset: an enlarged part of the graph). **h** Bar chart of reconstructed time on different FOVs. The loading of training weights for the first frame takes time. Two comparisons are conducted, without the first frame (upper row) and with the first frame (bottom row). Scale bar: 10 μm

We first verified the feasibility of RTU-Net in tubulin imaging and compared the performance of RTU-Net with other existing methods, including physics-based methods, such as VsLFM^10^ and light-field deconvolution (LFD)^1^, and end-to-end networks, such as HyLFM-Net^9^ and VCD-Net^3^. For consistent comparison, we utilized the high-resolution 3D data reconstructed by scanning light-field microscopy (sLFM)^19^ as the reference (Figs. 2a and S4) and generated the training and validation dataset by projecting the high-resolution 3D data into 2D light-field images. All networks were evaluated using the same new samples that were not part of the training or validation dataset. The results showed that RTU-Net successfully reconstructed the synthetic tubulins with the highest peak signal-to-noise ratio (PSNR), lowest learned perceptual image patch similarity (LPIPS), and mean squared error (MSE). The average PSNR of RTU-Net reached 41.22 dB (Figs. 2b and S4), while the value for other methods was lower than 40 dB. The average LPIPS of RTU-Net reached 1.882 × 10^−6^ (Fig. S5a), whereas the LPIPS of other methods was higher than 2.4 × 10^−6^. The average MSE of RTU-Net reached 8.373 × 10^−5^ (Fig. S5b), which was much lower than the reconstruction results of the other methods. In addition, RTU-Net provided the lowest mean value (0.0011) for the difference map of tubulins (Fig. S5c), which was much lower than other methods (0.0035–0.0006). To further compare the reconstruction results, the 3D volumes reconstructed by all five methods were maximum intensity projected (MIP) onto the x-y and x-z planes, respectively (Figs. 2c, S4, and Video 1). The MIP images demonstrated that RTU-Net reconstructed synthetic tubulins with minimal artifacts. In contrast, VsLFM incorrectly recreated multiple non-existent structures (highlighted with blue lines), and both HyLFM-Net and VCD-Net failed to recover the features labeled with green lines. In addition, due to the low resolution, the image produced by LFD presented the tubulin structure in a thicker manner than the actual structure. The outstanding performance of RTU-Net was further confirmed in the enlarged view of the MIP image, which clearly depicted the artifacts and ghosting produced by other approaches (Fig. 2d). Subsequently, we performed a frequency analysis on images reconstructed by all the methods. Our findings revealed that the Fast Fourier Transform (FFT) of the image reconstructed by RTU-Net exhibited a spectrum that closely resembled that of the ground truth (Fig. 2e), with a structural similarity index measure (SSIM) value of 0.8455 when compared to the spectrum of ground truth. In contrast, the spectra of the other methods were noticeably distinct from that of the ground truth, with SSIM values much lower than that of RTU-Net. To further compare the specific details reconstructed by different methods, we chose one of the reconstructed proteins and conducted a normalized intensity analysis. The findings suggest that the structure reconstructed by RTU-Net had an intensity distribution that was closest to that of the ground truth (Fig. 2f), while the intensity distribution obtained by other methods either failed to resolve the peak or revealed redundant features due to the poor resolution.

We also tested the performance of RTU-Net in the reconstruction of tubulin with variant densities and diameters, and compared results with that of other end-to-end networks, including VCD-Net and HyLFM-Net (Figs. S6 and S7). For the test of densities, the images of tubulin with 5 densities were tested. The results indicated that the SSIM between the output of each network and ground truth decreased as the density of tubulin increased, but RTU-Net attained the best results when the density remained constant (Fig. S6). For the test of diameters, we reconstructed 10 μm-diameter synthetic tubulins in addition to the 1 μm-diameter structure with all networks. Quantitative analysis revealed that the Pearson correlation and SSIM of RTU-Net were close to 1, and the PSNR of RTU-Net was as low as 35 dB, which was much better than other methods (Fig. S7).

We then quantified the prediction speed of RTU-Net for volumes with sizes ranging from 35 × 35 × 12 μm^3^ to 210 × 210 × 12 μm^3^, using 16 frames of images for each test. The results showed that the prediction time of the first frame increased from 0.38 s (35 × 35 × 12 μm^3^) to 1.56 s (210 × 210 × 12 μm^3^) as the size increased. However, the prediction time required after the prediction of the first frame was stable at ~0.004 s, with minimal relationship with field-of-view (FOV) (Fig. 2g and h). The 0.004 s prediction speed equals 250 Hz volume rate, enabling RTU-Net for real-time light-field image reconstruction.

### RTU-Net demonstrated superior resolution in reconstructing microbeads

We then verified the feasibility of RTU-Net through the reconstruction of microbead with a diameter of 100 nm. Results demonstrated that RTU-Net obtained the best resolution among all the tested networks (Fig. S8). Specifically, RTU-Net accurately resolved a small particle that was invisible for other methods (Fig. S8a, bottom right); RTU-Net was also the only network that distinguished two closely located (199 nm) particles (yellow arrow) (Fig. S8b), clearly showing two peaks in the profile of the beads. Quantitatively, RTU-Net attained 341.7 ± 60.65 nm and 271.5 ± 48.91 nm in axial and lateral full width at half maximum (FWHM), respectively, which were the best resolution among all the methods (Figs. S8c and S9). In terms of reconstruction efficiency, though VsLFM obtained a lateral resolution comparable to RTU-Net, the reconstruction time of VsLFM ( ~ 1600 s) was severely longer than that of RTU-Net ( ~ 0.004 seconds after the first frame) (Table S4) when tested in our server (Table S5). Considering both resolution and efficiency, RTU-Net is the best against all other reported networks for light-field reconstruction.

### RTU-Net demonstrated strong generalization ability under different microscale training samples

Generalization ability is critical in the application of deep learning in biological sample reconstruction, particularly in cross-sample experiments with diverse data. Collecting large datasets that cover a wide range of biological phenomena, such as specific types of cells in different physiological and pathological states, is time-consuming and extremely difficult. Therefore, developing a network that can maintain constant performance across the reconstruction of different biological samples is highly necessary. Previously reported networks, such as VCD-Net and HyLFM-Net, have been testified for weak generalization ability due to poor adaptation of the loss function and network structure^10^. Although later reported VsLFM improved generalization ability, the iterative computation required for volumetric reconstruction prohibited it for rapid and instant imaging. RTU-Net has the capability to surpass all prior deep learning methods in terms of generalization for light-field image reconstruction.

**Fig. 3:**
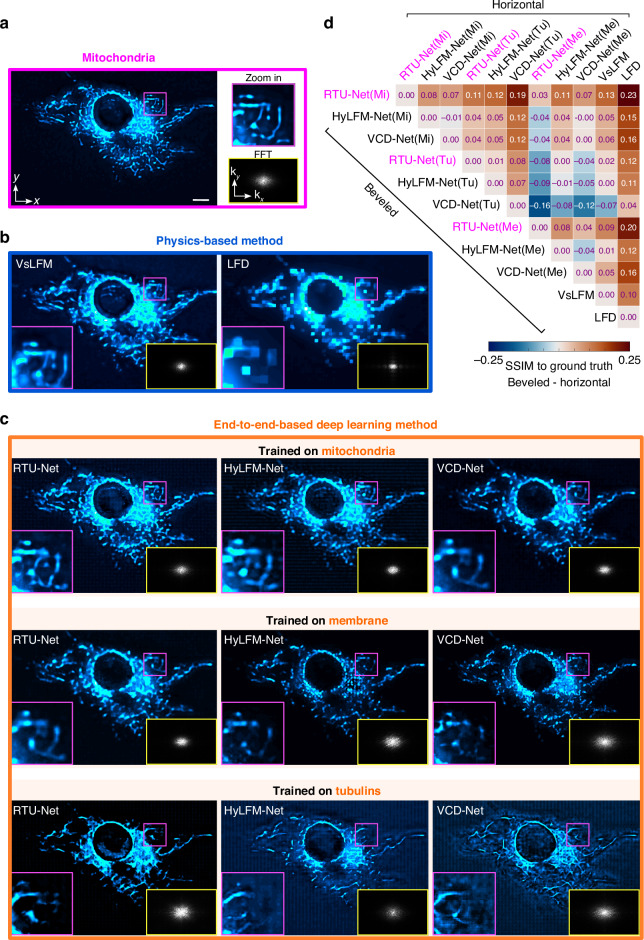
The generalization ability of RTU-Net with different training samples. **a** The ground truth MIP of a fixed L929 cell with mitochondria, acquired by sLFM with a ×63/1.4 NA oil-immersion objective in ideal imaging condition. The Fourier transform for the entire field of view is shown in the bottom right panel. The magnified view of the purple area is displayed at the top right panel. **b** MIPs of a fixed L929 cell with mitochondria reconstructed by VsLFM and LFD. The corresponding Fourier transform and enlarged views are shown at the bottom of each panel. **c** MIPs from a fixed L929 cell with mitochondria reconstructed by RTU-Net, HyLFM-Net, and VCD-Net. These neural networks were trained on different samples, including mitochondria, membranes, and tubulins. The Fourier spectrum and the enlarged view corresponding to each panel are shown at the bottom. **d** SSIM of mitochondria obtained by RTU-Net (trained on mitochondria), HyLFM-Net (trained on mitochondria), VCD-Net (trained on mitochondria), RTU-Net-Net (trained on membrane), HyLFM-Net (trained on membrane), VCD-Net (trained on membrane), RTU-Net (trained on tubulins), HyLFM-Net (trained on tubulins), VCD-Net (trained on tubulins), VsLFM and LFD. The positive values indicate that the beveled method has a higher SSIM compared to the horizontal method, whereas the negative values indicate the opposite. Scale bar:10 μm

In order to validate the exceptional effectiveness of RTU-Net, we conducted a comparative analysis of RTU-Net, VsLFM, VCD-Net, HyLFM-Net, and LFD using cell imaging techniques. Specifically, we focused on the mitochondria, which played a crucial role in respiration and cell signaling pathways, and the cell membrane, which was essential for energy production and signaling processes within the cell. Hence, volumetric and high-resolution reconstruction of mitochondria and membranes is highly wanted to investigate cellular bioenergetics and cell signaling. In this test, the mitochondria and membrane reconstructed through scanning light-field microscopy (sLFM) were used as the ground truth (Figs. 3a and S10a and S11a), and then the same sample was reconstructed through several methods such as physics-based networks, i.e., VsLFM, and LFD, and end-to-end networks, i.e., RTU-Net, VCD-Net, HyLFM-Net. Compared to ground truth, images reconstructed by physics-based methods revealed obvious defects. The image reconstructed by VsLFM showed aberrations, leading to a loss of structures (labeled with the purple box); the image reconstructed by LFD was blurry as a result of low resolution (Figs. 3b and S10 and S11).

We then trained RTU-Net, HyLFM-Net, and VCD-Net on mitochondria, membrane, and tubulins, respectively, and tested these networks on mitochondria. When both the test dataset and the training dataset were based on mitochondria, all three networks demonstrated good refactoring performance. However, when these network models were trained on membrane and tubulin data and tested on tubule data, the performance of VCD-Net and HyLFM-Net degraded significantly. In contrast, RTU-Net achieved consistent results even when the training data set was changed, suggesting the strongest generalization ability over other networks (Figs. 3c and S11f).

To provide a clearer quantitative assessment of the performance of each method, we calculated the SSIM between ground truth and the reconstructed volumes obtained by each method, respectively. We arranged these methods based on the order of beveled and horizontal edges (Fig. 3d), and subtracted the SSIM of the horizontal edge from the SSIM of the beveled edge method, yielding the corresponding value on the heat map. The positive subtraction result indicated that the SSIM value of the beveled edge method was higher than that of the corresponding horizontal edge method, and vice versa. Expectedly, the sample SSIM of RTU-Net trained by mitochondria was higher (0.07–0.23) than that of all the other methods. In contrast, SSIM value of VsLFM was above that of VCD-Net trained solely on tubulins. Again, this experiment has successfully demonstrated the superiority generalization of RTU-Net.

### RTU-Net facilitated accurate quantitative analysis of neural activity in microscale zebrafish light-field imaging

We then examined RTU-Net’s performance on the reconstruction of overtime data, which was the 10 Hz light-field image of zebrafish larvae brain expressing GCaMP6. For comparison, we reconstructed the data with RTU-Net, VCD-Net, and HyLFM, and extracted the activity of three neurons from the same position for three networks (Fig. S12). The results showed that the neural signal obtained by RTU-Net was almost the same as the true values, while VCD-Net and HyLFM failed to fully recover the neural activity. This validation successfully proved the superior dynamic volume imaging capability of RTU-Net.

### RTU-Net demonstrated accurate analysis of neural activity in mesoscale light-field imaging

**Fig. 4:**
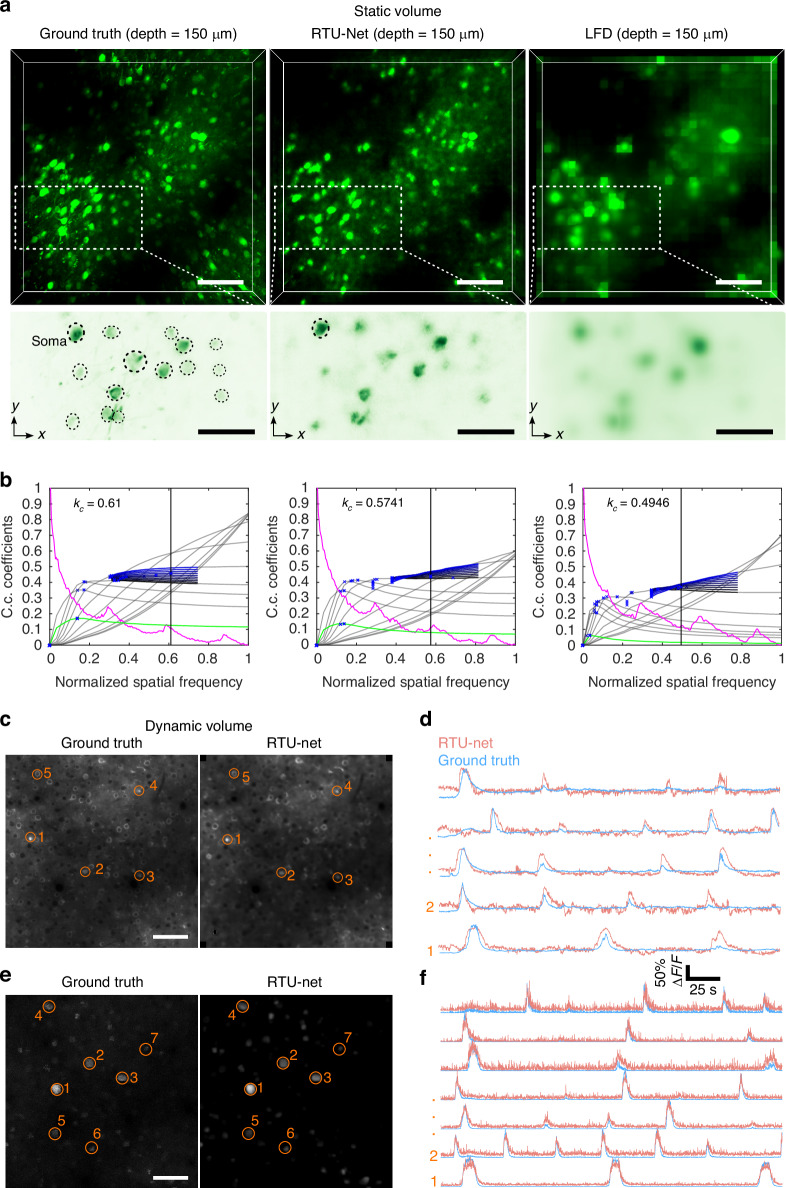
RTU-Net performance on in vivo two-photon imaging data. **a** 3D rendering static volumes and enlarged MIPs of neurons at a depth of 150 μm in mouse cortex, obtained by OLYMPUS cellSens Dimension (left), RTU-Net (middle), and LFD (right). **b** Decorrelation functions and the estimated normalized cut-off frequency *k*_*c*_ for ground truth, RTU-Net, and LFD. Gray curves represent decorrelation functions with high-pass filtering; green curves represent the decorrelation function without any high-pass filtering; vertical black lines represent the cut-off frequency. **c** Comparison of dynamic volumetric light-field reconstruction methods and ground truth. Simulated mouse primary visual area V1 ground truth volume (depth = 0 μm) containing neurons, blood vessels, and neuropil (left). RTU-Net reconstructed volume (right). **d** Neuronal activity traces corresponding to circles in (**c**). **e** Simulated ground truth volume (depth = 0 μm) only containing neurons (left) and the RTU-Net reconstructed volume (right). **f** Neuronal activity traces corresponding to circles in (**e**). Scale bar: 20 μm for (**a**) (top), (**c**, **e**), and 50 μm for (**a**) (bottom)

LFM is a promising method for neural activity imaging in small model organisms. To demonstrate the potential of RTU-Net for mesoscale light-field image reconstruction, we reconstructed structural and functional neuron images of mouse brain at mesoscale. We used a two-photon microscope (Olympus FVMPE-RS) to acquire the high-resolution 3D structural image of a mouse brain labeled by green fluorescence protein and then back-projected the two-photon volume into a light-field image. Following that, we reconstructed the light-field image with RTU-Net and the conventional method and compared the reconstructed results with the two-photon image (ground truth). The results indicated that RTU-Net has recovered a volume comparable to ground truth (Fig. 4a top and Video 2), and can obviously resolve soma at a depth of 150 μm with more elaborate detail than LFD (Fig. 4a bottom), allowing accurate localization of soma (Fig. S13). We further confirmed the superior resolution of RTU-Net by quantifying the cut-off frequency^20^. We input 3D volume subjected to a total of 10 high-pass filters (from weak to strong filtering) and then obtained the normalized cut-off frequency (k_c_) (Fig. 4b) across all depths, which was 0.5741 for RTU-Net, but was 0.4946 for LFD, indicating higher resolution of RTU-Net.

We then tested RTU-Net through simulated mesoscale functional neural imaging of brain activity reported by nuclear-confined calcium indicator GCaMP6s^21,22^. Using the method reported by NAOMi^22^, we conducted a simulation of two distinct types of mouse brain cortex, with a volume acquisition rate of 10 Hz. One of the simulations included neurons, blood vessels, and neuropil in the visual 1 area, whereas the other simulation just included neurons. The ground truth was the simulated high-resolution image, whereas the light-field image was generated using back projection. We first reconstructed the light-field volume with RTU-Net and then extracted the neural activity (Fig. 4c–f). While the RTU-Net reconstructed neural image closely resembles the ground truth image when blood vessels and neuropil were taken into account during simulation, the extracted neural activity differed significantly from the ground truth (Fig. 4c, d). This discrepancy was mostly caused by the strong interaction of background. However, when the background was removed from the simulated data, both the reconstructed neural image and the neural activity of RTU-Net approximated that of the ground truth (Fig. 4e, f), successfully proving the efficacy of RTU-Net for mesoscale light image reconstruction.

### RTU-Net provided high-resolution particle localization in macroscale light-field PIV

**Fig. 5:**
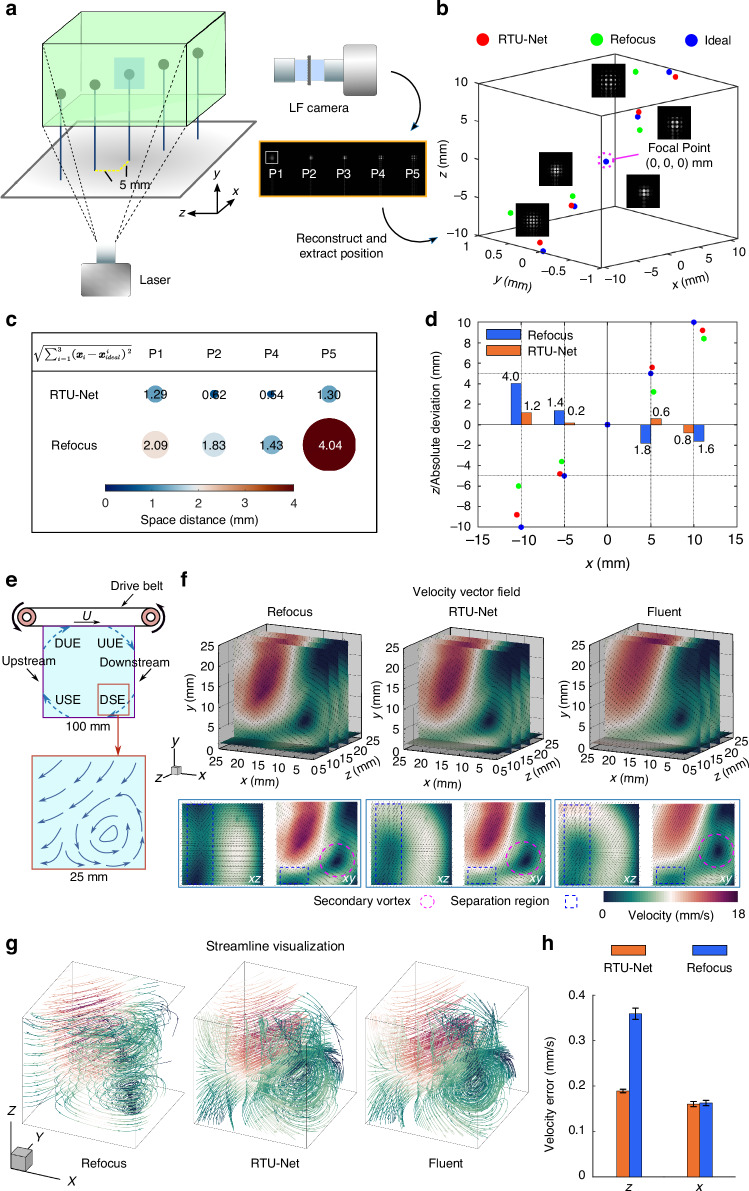
**The performance evaluation of RTU-Net on LF-PIV imaging of square lid-driven cavity flow.** **a** The diagram illustrates an experiment with five particles placed vertically, spaced apart by 5 mm intervals. **b** The light-field image captured in (**a**) is reconstructed by refocusing (green) and RTU-Net (red), respectively. The central position of each particle is extracted, in which blue is the ideal position. **c** In Cartesian coordinates, the distance between the reconstructed particle position **x**_**i**_ and the ideal position ${{\boldsymbol{x}}^{i}_{ideal}}$ in space is expressed as a tensor ${\sqrt{{\sum}^{3}_{i=1}\left({{\boldsymbol{x}}_{i}-{\boldsymbol{x}}^{i}_{ideal}}\right)^2}}$. **d** The histogram shows the absolute deviation between the particle’s actual and ideal location in the z direction from the perspective of x-z. **e** Schematic drawing of setups for square lid-driven cavity flow measurement (upper) and the secondary vortex in the imaging area (bottom). **f** The flow velocity vector fields acquired by refocusing, RTU-Net, and Fluent. The central point of the captured secondary vortex is marked by a purple circle, while the flow separation region is indicated by a blue box. **g** The streamline visualization derived from refocusing, RTU-Net, and Fluent. **h**, The velocity error provided by refocusing and RTU-Net when compared with Fluent-simulated velocity vectors, n = 5000

Following the previous tests, we examined the performance of the RTU-Net in reconstructing macroscale light-field PIV image^6,23,24^. PIV utilizes a laser to illuminate particles that have been seeded into the flow, then images these particles’ location with a camera at two successive frames. The particle images are divided into small interrogation windows to computationally determine the displacement of each window through optical flow or correlation algorithms^25,26^. The velocity vectors of the flow field are then calculated with the displacement and the time within which the displacement occurs. Apparently, accurate localization of particle position is essential to the measurement result. However, recently emerged LF-PIV has difficulty to preciously localize particle position due to the poor axial resolution. Considering the impressive results achieved by RTU-Net in reconstructing light-field images at both microscale and mesoscale levels, we are confident that RTU-Net can enhance the axial resolution for LF-PIV. To demonstrate the feasibility, we utilized RTU-Net to reconstruct a large-scale LF-PIV image. We first tested RTU-Net’s performance through the imaging of fixed particles. Practically, five particles (500 μm in diameter) were fixed at an interval of 5 mm between the x and z planes, and imaged with a customized light-field camera (Methods) (Fig. 5a). After reconstructing the light-field image, we extracted the central position of each particle and used the intermediate particle P3 as a reference to calculate the particle’s true position (Fig. 5b). Then we quantified the absolute error of the position tensor between the true position of the particle and the computed position which was obtained through the conventional refocusing algorithm and RTU-Net, respectively. The results demonstrate that the location estimated by RTU-Net exhibited minimal mismatch (mean: 0.94 mm) in comparison to the actual position. In contrast, the location estimated by the conventional refocusing approach^27^ exhibited severe inaccuracies (mean: 2.3 mm) (Fig. 5c). We also quantitatively computed the absolute error along the axial direction. The spatial deviation in our approach was 3 to 4 times greater than that of RTU-Net, especially at locations close to the edge. The main reason for these inaccuracies was the incorrect reconstruction of positions in the z-direction. At P2, the deviation was 7 times greater than that of our method. (Fig. 5c, d).

To demonstrate the superior performance of RTU-Net in imaging with LF-PIV, we conducted experiments on square lid-driven cavity flow (Method). We performed dynamic imaging over volume size of 90 × 40 × 40 mm^3^ and 25 × 25 × 25 mm^3^ in the downstream secondary vortex (DSE) when Reynolds number (Re) was 13144 and 3286, respectively, where the DSE was caused by the main flow separation in the angular region (Fig. 5e). We performed flow validation for the flow with Re = 13144 on different imaging sources (Fig. S14 and Videos 3 and 4), and the results showed that RTU-Net can be applied to dynamic reconstruction for different imaging sources. For the flow with Re = 3286, we reconstructed the 3D particle field using Refocusing^27^ and RTU-Net, respectively, and then calculated the velocity vectors of the reconstructed 3D particle field for two consecutive frames by the optical flow algorithm^28,29^ (Fig. 5f). For comprehensive comparison, we also computational simulated the lid-driven cavity flow with commercial software (Ansys Fluent) as a reference. The comparison of velocity fields at positions *y* = 1.5 mm and *z* = 7.5 mm indicated that the flow field computed based on the particle field reconstructed by the refocusing method differed significantly from the simulated results in the x-z plane, failing to reconstruct the separation structure (blue boxed labeled region in Fig. 5f) of the flow. However, the same separation region was successfully recovered when the particle field was reconstructed by RTU-Net. We also computed the 3D streamlines for the imaging volume. Again, the flow reconstructed using the refocusing approach showed minimal flow in the z direction due to its poor axial resolution (Fig. S15a and Video 4), while the flow field reconstructed by RTU-Net closely matched the Fluent simulation result (Fig. 5g), revealing a vortex shape. In addition, the velocity errors of flows also provided the superior performance of RTU-Net: the velocity vector reconstructed with particle field produced by RTU-Net demonstrated a much smaller error than the refocusing method. Although both velocity vectors revealed similar errors along the x-direction, they represented significantly different error distributions along the axial direction. The velocity errors for the refocusing method (0.3603 ± 0.0041 mm/s) reconstructed particle field were 86% higher than that obtained with RTU-Net (0.1934 ± 0.001 mm/s) (Fig. 5h and Fig. S15b).

**Fig. 6:**
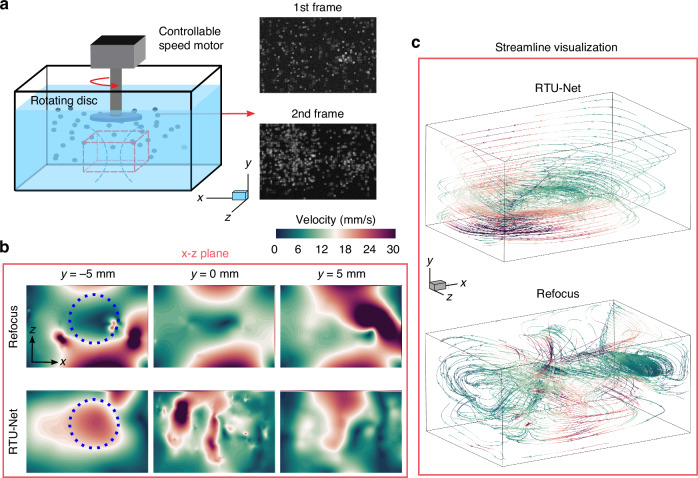
The performance evaluation of RTU-Net on LF-PIV imaging of vortex flow. **a** Schematic drawing of setups for vortex flow measurement. Two example frames are shown in the right. **b** Comparison of the flow field in the x-z plane at −5 mm, 0 mm, and 5 mm reconstructed by refocusing algorithm and RTU-Net. Blue dashed circles label a vortex, which was recovered by RTU-Net, but not recovered by conventional method. **c** The streamline obtained with refocusing algorithm and RTU-Net

We also tested the RTU-Net in vortices generated by a rotating disk, where the vortex axis was perpendicular to the axis of the imaging system, so high axial resolution was required to fully resolve the vortex profiles across the axis. The vortices were generated by rotating fan blades driven by a controlled-speed motor, which were controlled to rotate at low speeds, and the experiments were performed at three speeds of 9, 12, and 15 rpm (Fig. S16 and Video 5). Once the flow was stabilized, we irradiated the particles with a laser and used a common industrial camera to photograph the vortices within the flow region at a frequency of 40 Hz in order to reconstruct the flow’s three-dimensional structure at a finer level. The tracer particles used in the experiments were white polyethylene microspheres with a diameter of 255 µm and a particle concentration of 0.03 particles per microlens (PPM), and the overall cavity was a 200 × 100 × 150 mm^3^ acrylic five-sided box-shaped tank with an actual imaging area volume of 25 × 15 × 24 mm^3^. The imaging area had a volume of 25 × 15 × 24 mm^3^. We also used a light-field camera to capture two consecutive images of the flow field (Fig. 6a), and then reconstructed the flow field using the optical flow method and compared the results of the RTU-Net and refocusing method. Our findings showed that RTU-Net has successfully resolved the complete vortex structure from the velocity profiles and the streamline images, due to the high axial resolution reconstruction (Fig. 6b, c). In contrast, the refocusing method cannot accurately estimate the axial distribution of the particles, leading to disordered flow vectors and streamlines (as shown in Fig. 6b and c). Therefore, RTU-Net shows promise as a general tool for studying macroscale flow measurements at high fidelity.

### RTU-Net proved strong generalization ability in 5-round validation and in reconstruction of data from variant imaging sources and scale

The generalization of RTU-Net is essential for its application in a broad domain, especially when the data scale and imaging sources change. We first performed 5-round validation (leave-one-out method) to test the generalization of RTU-Net (Table S6). Specifically, we split a dataset of 2000 data into 5 portions, and in each round we used 4 portions (1600 data) for training and the other 1 portion (400 data) for validation. The testing dataset was the same during the 5 rounds and was different from the training and validation data. In the performance evaluation, the values of PSNR, SSIM, LPIPS, MSE and Pearson correlation have a small gap between different rounds and showed high consistency. For example, PSNR ranged from 39.03 to 40.46 and SSIM ranged from 0.9738 to 0.9802 with small fluctuation, indicating that the model’s performance was stable. This indicated that the model has good generalization ability and its prediction results have high reliability under different training data sets.

We then further tested the generalization ability of RTU-Net on data from different sources (Figs. S14 and S17) and different scales (Fig. S17), both of which were unseen data to RTU-Net. We have classified our tests into three categories, one was the test for images from the same imaging source but at different scales, which was conducted with microtubulin data, and another was the test for images from different imaging sources, which was tested with micro tubulin data and macroscale LF-PIV data. We then quantified the generalization performance of RTU-Net with PSNR, SSIM, and LPIPS.

For the same imaging source test, we took 10 μm tubulins as the example, generated the dataset, and trained RTU-Net at 40× magnification. Then we predicted the unseen data that was not included in the training set at10×, 19.4×, 40×, 63× magnification, respectively. The results indicated that, even when the scales varied largely from 10× to 63×, RTU-Net’s PSNR, SSIM, and LPIPS remained almost constant, demonstrating that RTU-Net has strong generalization ability for unseen data at different scales (Fig. S17a–e).

For the different imaging source tests with tubulin, we generated a dataset with 10 μm tubulin, trained RTU-Net on imaging source 1 at 40× magnification, and then predicted the unseen data from imaging source 2 that has different sizes in microlens. The predicted data was not included in the training set and was magnified by 63×, 40×, 19.4×, and 10×, respectively. Even when the network was trained on 40× data from imaging source 1, RTU-Net was still able to reconstruct light-field image at different scales from imaging source 2, by providing PSNR, SSIM, and LPIPS that were only slightly worse than that obtained with data from imaging source 1. The overall reconstruction results were still close to the true value (Fig. S17f–i). This test successfully demonstrated that the RTU-Net has a strong generalization ability for different scales of imaging under different imaging sources.

For the different imaging source tests with LF-PIV, we assessed RTU-Net’s generalization ability on the reconstruction of LF-PIV images from different imaging sources in macroscale. Firstly, we used RTU-Net to reconstruct LF-PIV images of top cover-driven square-cavity flow in a 90 × 40 × 40 mm^3^ transparent square chamber (Fig. S14a). The Reynolds number of the flow was 13144. The images were two different imaging sources at 40 Hz with different microlens sizes (Fig. S14b). When the network was trained on data from imaging source 1, it can reconstruct the data from both imaging sources and can localize particles clearly (Fig. S14c), and Video 4), demonstrating the strong generalization of RTU-Net.

## Discussion

To summarize, we have demonstrated a deep learning-based method that can reconstruct high-resolution light-field images at the microscale, mesoscale, and macroscale levels. At the microscopic scale, we reconstructed tubulins, mitochondria, and mitochondrial cell membranes and demonstrated that RTU-Net has the highest spatial resolution and minimal reconstruction artifacts compared to reported networks. At the mesoscopic scale, we reconstructed mouse cortex volume, extracted neurons for activity trace analysis, and obtained traces that highly resemble ground truth. At the macroscopic scale, we performed LF-PIV measurements, and the results showed that the RTU-Net proved 3 to 4 times improvement in particle localization resolution and thereby allowing high-quality reconstruction of flow dynamics. Furthermore, RTU-Net exhibited superior generalization capability compared to previous end-to-end networks, and achieved a reconstruction speed that was 3.2 × 10^5^ times (light-field image of the same size: 1248 × 1248 pixels) faster than physical iteration-based reconstruction approaches. Quantitatively, RTU-Net outperformed conventional methods in a number of metrics. It provided the best PSNR, LPIPS, and the lowest MSE among all the compared methods, showing excellent robustness and reconstruction accuracy (Table S3). As a network that bridges the reconstruction of light-field images from the microscale to the macroscale, RTU-Net has successfully broken the limitation of other reported methods that are only demonstrated in the microscale.

Because one advantage of RTU-Net is that it can be trained on small datasets, potential overfitting to synthetic datasets could be prevented by continuously reducing the data size for synthetic or simple datasets. However, the current RTU-Net still has some limitations. Firstly, RTU-Net can only be applied to light-field imaging, which can be subsequently compensated by appropriate adjustments to the network structure. However, its underlying mechanism can be extended to various imaging techniques that utilize iterative or trained computational approaches for image reconstruction or recovery. Secondly, for real experimental data with noise, the incorporation of a denoising module into RTU-Net might be necessary. Thirdly, the training time of the network is slightly long and the resolution could be further improved, which can be achieved by utilizing more powerful graphics processing units and better pre-processing/pre-training strategies, respectively. Finally, the volume size of RTU-Net reconstruction is dependent on the graphics memory, which can be subsequently solved by making the model more lightweight or using more advanced image coding methods.

Ultimately, we expect that RTU-Net can bring new insights into computational imaging techniques, as it has successfully demonstrated the ability to reconstruct multiscale light-field images at high-resolution with constant performance. We envision that RTU-Net provides more possibilities for us to explore large-scale imaging that can further enhance our understanding of science.

## Materials and methods

### Dataset preparation

At the microscale, we prepared samples (64×, 40×, and 19.4×) through two strategies (Tables S7 and S8). First, when the performance comparison includes VsLFM, we chose to use the dataset of VsLFM for training for all the reconstruction methods, considering the difficulty in obtaining scanning light-field image data due to the lack of scanning light-field equipment^10^. In this case, the ground truth was the volume obtained after processing scanning light-field images with sLFM. Second, for the comparison without VsLFM, we used the VCD-LFM dataset (zenodo.org) for training. At the mesoscale, we simulated the volume of neural activity in the rat cerebral cortex with the mesoscopic field imaging parameter 10×/0.4 NA objective (Table S9) for mesoscopic samples training^30^. At the macroscale, we trained macroscopic particle samples by simulating particles with 0.5×/0.045 NA objective (Table S10).

### RTU-Net architecture

Our network was trained to learn the mapping relationship between the raw light-field image and the 3D depth images (Y). The problem can be written as:

1 $$Y=f\left({LF};\theta \right)$$

where LF indicated light-field image, θ represented the network parameters to be learned through training and Y represented the function that maps from light-field image to 3D volume.

The generator (G) of RTU-Net was based on an encoder-decoder structure. The encoder was a feature extraction module that took the multi-view sub-images of the raw light-field images as input and consisted of three convolutional blocks, including 3 × 3 kernel, batch normalization, and ReLU. The decoder employed the same structure as the encoder. It should be noted that we improved the skip connections. Specifically, we determined the optimal connection through ablation experiments (Table S11) by simply connecting the even-channel portions of the encoder and decoder in tandem at layers 1 and 3, respectively, which yielded better results than the traditional skip-connection approach. In addition, to suppress the edge serrated and checkboard artifacts that were induced by solely using the bicubic interpolation and the pixel shuffle checkerboard that existed during training^31^, we combined bicubic and pixel shuffle for sampling and proved that the artifacts can be significantly reduced.

The discriminator (D) of RTU-Net consisted of five convolutional blocks and two dense layers. Each convolutional block contains two convolutional layers, but the channel number applied in each layer differs for each block. For the first block, its layer’s channel number matched the slice number of the reconstructed volume. For the other blocks, the channel number used in each layer was 20 × 2^k^, where k was the layer number. A global average pooling layer was then applied to change the size of feature maps in each channel. The five convolutional blocks were followed by two dense layers (1 × 1 convolution). The first dense layer has 20 hidden units with ReLU activation function, and the second dense layer used a sigmoid activation function. The detailed structure of the GAN module was illustrated in Figs. S18, S19, Tables S12 and S13.

### Loss functions

The training loss of RTU-Net was composed of three parts: pixel-wise BerHu loss^32^, MS-SSIM^33^, and adversarial loss using the condition GAN (cGAN) structure^34^. In our study, the total loss of RTU-Net was expressed as:

2 $${\arg }\mathop{\min }\limits_{G}\mathop{\max }\limits_{D}{L}_{{\rm{GUR}}-{\rm{Net}}}=\alpha {L}_{\text{BerHu}}\left({\text{G}}\left(x\right),y\right)+\,\beta {L}_{\text{MS}-\text{SSIM}}\left({\text{G}}\left(x\right),y\right)+\gamma {L}_{\text{cGAN}}\left(\text{G},\text{D}\right)$$

where *x* was the input light-field image, and *y* was the ground truth. *α*, *β*, *γ* were coefficients. The best combination *α*, *β*, *γ* was determined through loss function weighting ablation experiments (Table S14). Specifically, the performance of each weight was validated with metrics such as PSNR, SSIM, and LPIPS. Our study indicated that when $\alpha$=3, $\beta$=1, $\gamma$=0.5, the network produced the best performance without blur, so we used this combination in the weight of our loss function.

The MSSSIM and BerHu losses are expressed as:

3 $$\text{MSSSIM}(\text{G}\left(x\right),y)={\left[\frac{2{\mu }_{\text{G}{\left(x\right)}_{M}}{\mu }_{{y}_{M}}+{C}_{1}}{{\mu }_{\text{G}{\left(x\right)}_{M}}^{2}+{\mu }_{{y}_{M}}^{2}+{C}_{1}}\right]}^{{\alpha }_{M}}\times\mathop{\prod}\limits_{j=1}^{M}{{\left[\frac{2{\sigma }_{\text{G}{\left(x\right)}_{j}}{\sigma }_{{y}_{j}}+{C}_{2}}{{\sigma }_{\text{G}{\left(x\right)}_{j}}^{2}+{\sigma }_{{y}_{j}}^{2}+{C}_{2}}\right]}^{{\beta }_{j}}\left[\frac{{\sigma }_{\text{G}{\left(x\right)}_{j}{y}_{j}}^{2}+{C}_{3}}{{\sigma }_{\text{G}{\left(x\right)}_{j}}{\sigma }_{{y}_{j}}+{C}_{3}}\right]}^{{\gamma }_{j}}$$

4 $${\rm{BerHu}}\left(G\left(x\right),y\right)\,=\sum _{\left|{{\rm{G}}\left(x\right)}_{\left(m,n\right)}-{y}_{\left(m,n\right)}\right|\le c}\left|{{\rm{G}}\left(x\right)}_{\left(m,n\right)}-{y}_{\left(m,n\right)}\right|+\sum _{\left|{{\rm{G}}\left(x\right)}_{\left(m,n\right)}-{y}_{\left(m,n\right)}\right| > c}\frac{[{{\rm{G}}\left(x\right)}_{\left(m,n\right)}-{y}_{\left(m,n\right)}]^{2}+{c}^{2}}{2c}$$

where $\text{G}{\left(x\right)}_{j}$, ${y}_{j}$ were the 2^*j*-1^ downsampled images of *x* and *y*, respectively. ${\mu }_{{y}_{M}}$ was the mean of *y*, ${\mu }_{\text{G}{\left(x\right)}_{M}}$ was the mean of ${\rm{G}}\left(x\right)$, ${{\rm{\sigma }}}_{y}^{2}$ was the variance of *y*, ${{\rm{\sigma }}}_{{\rm{G}}\left(x\right)}^{2}$ was the variance of ${\rm{G}}\left(x\right)$, and ${{\rm{\sigma }}}_{{\rm{G}}\left(x\right)y}^{2}$ was the covariance between $\text{G}\left(x\right)$ and *y*, ${{\rm{G}}\left(x\right)}_{\left(m,n\right)}$ was the intensity value at the pixel (*m*,*n*) of image ${\rm{G}}\left(x\right)$, and *y* was the intensity value at the pixel (*m*,*n*) of image ${y}_{\left(m,n\right)}$. ${\alpha }_{M}$, ${\beta }_{j}$, ${\gamma }_{j}$, ${C}_{i}$ were empirical constants^35^ and *c* was a constant (set as 0.1). BerHu loss addresses robustness to outliers in tasks, and MSSSIM loss focuses on capturing perceptual differences in image similarity assessment. Unlike the previous loss function that only recognized overall or average pixel values, the adversarial loss function emphasized the high-level features of the image, such as texture features, shape features, and spatial relationship features at a single pixel scale.

The combination of MSSSIM, which was used to evaluate regional/global similarity, and a pixel-wise loss term (such as L1, L2, Huber, and BerHu), which was utilized to evaluate the visual quality and preservation of fine details between restored image and the ground truth, has demonstrated improved performance in translating and restoring image^36^.

For GAN network, we incorporated an adversarial loss function. The adversarial loss function was defined as:

5 $${L}_{\text{cGAN}}\left(\text{G},\text{D}\right)=\frac{1}{2}{\text{E}}_{\text{G}(\text{x})}\left[\log \left(1-\text{D}\left(\text{G}\left(\text{x}\right)\right)\right)\right]+\frac{1}{2}{\text{E}}_{\text{y}}\left[\log \left(\text{D}\left(\text{y}\right)\right)\right]$$

### Data preprocessing and training procedures

All networks were implemented in Python with the PyTorch framework. Our training procedure used the AdamW optimizer (β1 = 0.9, β2 = 0.999^37^) with weight decay to be 1 × 10^-2^ and the learning rates of the generator and discriminator to be 1 × 10^−6^ and 1 × 10^−7^, respectively. The learning rate was reduced by a factor of 0.95 for every 5 calendar elements, and the batch size was 2 (Table S15). Meanwhile, we used alternating discriminator and generator training to train the network. To prove the converge performance of our loss function, we have performed ablation test, and compared the loss decay curve of our loss function with that of MSE, MS-SSIM, BerHu, MAE, and the combination of BerHu and MS-SSIM, respectively (Figs. S20, S21 and Table S16). The results showed that the generator loss of our network converged close to 0, which successfully demonstrated RTU-Net’s good convergence performance.

The time consumption for the RTU-Net procedure depended on the dataset size and computational resources. As a reference point, RTU-Net reached convergence after 400 calendar hours of training on 2164 pairs of synthetic tubulin image patches normalized to (−1, 1), each pair containing a light-field image (208 × 208 pixels) and a volume (208 × 208 × 61 pixels). The normalized formula was:

6 $${nomarlize}\left(x\right)=2\times \frac{x-\min \left(x\right)}{\max \left(x\right)-\min \left(x\right)}-1$$

where *x* was the image (light-field image/volume) to be normalized, min (∙) computed the minimum value of the input image, and max (∙) computed the maximum value of the input image. This meant that our RTU-Net can be trained without large dataset size, which was one of the advantages of our network. To validate this point, we trained our network with larger dataset sizes, predicted data that was not included in the training dataset with the trained network, and then quantitatively assessed the reconstructed image. The results revealed that when the training dataset size varies from 2000 4000 to 6000, parameters such as PSNR, SSIM, LPIPS, MSE, and Person correlation only changed slightly (Table S17), demonstrating that small training dataset size worked well for our network and no overfitting occurred. The results also proved that our network was cost-effective. The computation was performed on a workstation equipped with Intel(R) Xeon(R) Platinum 8269CY CPU @ 2.50 GHz and A100 PCIe 40GB graphic card.

### Optimizer ablation experiments

We performed ablation experiments on the network with different optimizers (Table S18), batch sizes (Table S19), and learning rates (Table S20). Among the optimizers, Rprop, SGD, and AdamW were chosen. The results showed that the PSNR using AdamW was 5.11 dB higher than that of Rprop and was 14.08 dB higher than that of SGD. Similarly, the SSIM using AdamW was 0.0292 and 0.9112 higher than that of Rprop and SGD, respectively. Moreover, AdamW also attained the lowest LPIPS and the Pearson correlation among the three optimizers, clearly demonstrating the advanced performance of AdamW.

### Batch size ablation experiments

For the ablation experiments with different batch sizes, we chose batch sizes of 2, 4, 8, and 16 (Table S19), and the results showed that the PSNR of different batch sizes only differed by less than 0.6 dB, the SSIM differed by less than 0.01. Also, the LPIPS differed by a maximum of 3.02 × 10^−7^, and the MSE differed by a maximum of 1.356 × 10^−5^, respectively. The maximum difference in Pearson correlation was 0.0269, which indicated that RTU-Net can adapt to different batch-size training and has little effect on the reconstruction results.

### Learning-rate ablation experiments

We chose the generator/discriminator learning rates of 1 × 10^−5^/1 × 10^−6^, 1 × 10^−6^/×10^−7^, and 1 × 10^−7^/1 × 10^−8^ for the ablation experiments (Table S20), and the results showed that the network was optimal when the generator/discriminator learning rate was 1 × 10^−6^/1 × 10^−7^. Concurrently, the loss decline curve of RTU-Net was plotted at varying learning rates (Fig. S22). When the learning rate was minimal, the convergence of the network was sluggish due to the limited step size, which can result in the network converging at a local extreme point, leading to the absence of a genuine optimal solution. Conversely, when the learning rate was substantial, the network converged more rapidly. However, the large step size can cause the network to depart from a specific optimal extreme point, thereby missing the identification of the optimal solution.

### Regularization method

Batch normalization was used in the network structure of RTU-Net, and we used the default weight decay of 1×10^-2^ by the AdamW optimizer during training. Dropout was not included for the following reasons. First, deep networks with residual connectivity tend to be more stable and less sensitive to overfitting^38,39^. Second, we have incorporated batch normalization into the network, which not only sped up training but also acted as a regularizer to some extent, reducing the dependence on dropout^40^. Continuing to include dropout may lead to a series of more problems such as model underfitting, difficulty in training convergence, and degradation of generalization ability. Third, our training used AdamW, which was an adaptive optimizer with weight decay and regularization that also reduced the dependence on dropout^41^.

### Performance metrics

We employed PSNR and SSIM to quantitatively evaluate the performance of RTU-Net. The formula for PSNR was:

7 $${\rm{PSNR}}=10\mathrm{lg}\frac{{MA}{X}_{I}^{2}}{{|\left|Y-X\right||}_{2}^{2}}$$

where X represented the ground truth, Y represented the corresponding reconstructed result, and MAX_I_ was the maximum pixel value of an image. MAX_I_ was 255 when each sampling point was represented by 8-bit. SSIM was calculated by the following formula:

8 $$\text{SSIM}=\frac{\left(2{{\rm{\mu }}}_{X}{{\rm{\mu }}}_{Y}+{\left(0.01\cdot \text{R}\right)}^{2}\right)\left(2{{\rm{\sigma }}}_{{XY}}+{\left(0.03\cdot \text{R}\right)}^{2}\right)}{\left({{\rm{\mu }}}_{X}^{2}+{{\rm{\mu }}}_{Y}^{2}+{\left(0.01\cdot \text{R}\right)}^{2}\right)\left({{\rm{\sigma }}}_{X}^{2}+{{\rm{\sigma }}}_{Y}^{2}+{\left(0.03\cdot \text{R}\right)}^{2}\right)}$$

where *X* was the ground truth signal, *Y* denoted the corresponding reconstruction result signals, *µ*_*X*_ and *µ*_*Y*_ represented the average values of each signal, *σ*_*X*,_ and *σ*_*Y*_ represented the corresponding variance of each signal, and *σ*_*XY*_ was the covariance for *X* and *Y*. *R* was the dynamic range and was 1 when the data was normalized to a single-precision floating-point number. LPIPS was realized by deep neural network; the specific architecture and parameters were obtained by large-scale training, which can be used to capture the perceptual information of the image. The calculation formula was as follows:

9 $${\rm{LPIPS}}={\sum }_{l}\frac{1}{{H}_{l}{W}_{l}}{\sum }_{h,w}{\left|\left|{w}_{l}\odot \left({\hat{X}}_{hw}^{l}-{\hat{Y}}_{{hw}}^{l}\right)\right|\right|}_{2}^{2}$$

where *X* denoted the ground truth signal, *Y* denoted the corresponding reconstruction result signal, *l* was the number of network layers of the network used for LPIPS, *w*_*l*_ was the weight of the *l*th layer of the network, *H*_*l*_ and *W*_*l*_ were the sizes of the images of the *l*th layer of the network, respectively, and ${{||}\bullet {||}}_{2}^{2}$ denoted the square of the L2 paradigm. The MSE was calculated as follows:

10 $${\rm{MSE}}={|\left|Y-X\right||}_{2}^{2}$$

where *X* denoted the ground truth signal, *Y* was the corresponding reconstruction result signal, and ${{||}\bullet {||}}_{2}^{2}$ was the square of the L2 paradigm.

We also used the Pearson correlation coefficient (ρ) to evaluate the similarity between the ground truth and the results obtained by different methods. ρ was calculated by the following formula:

11 $$\rho =\frac{\text{E}\left[\left(X-{\mu }_{X}\right)\left(Y-{\mu }_{Y}\right)\right]}{{\sigma }_{X}{\sigma }_{Y}}$$

where *X* and *Y* denoted the signals, *μ*_*X*_ and *μ*_*Y*_ represented the mean values of each signal, *σ*_*X*_ and *σ*_*Y*_ denoted the corresponding standard deviations of each signal, and E [∙] denoted the expectation.

### The analysis of neural activity

To quantify the fluorescence intensity of individual neurons, all pixels within a specific neuron’s region of interest (ROI) were averaged over time, resulting in a singular value denoted as *F*, representing the fluorescence intensity of that particular neuron. To well extract calcium dynamics, we computed neuron-specific baseline *F*_*0*_, which was determined as the average of all F recordings. The calcium dynamics were then extracted using the formula Δ*F/F*_*0*_ = *(F* − *F*_*0*_*) /F*_0_.

### Zebrafish imaging

Zebrafish larvae (5–7 days after fertilization) expressing GCaMP6 were imaged with scanning widefield microscopy (ground truth) and light-field microscopy. The light-field data was reconstructed by RTU-Net, VCD-Net and HyLFM-Net, and was then processed with CaImAn^42^ to extract the neural activity.

### CFD simulation of square lid-driven cavity flow

We used the commercial simulation software Ansys Fluent to carry out numerical simulation for square lid-driven cavity flow. The flow field volume was 100 × 100 × 100 mm^3^ and included 2 million grids. The flow was modeled by the κ-ω SST turbulence model, and the boundary layer’s grid encryption was used to make sure that y+ (the position of the mesh’s first layer in the boundary layer) was smaller than 1 in order to better represent the flow field structure close to the wall. The drive belt in the experiment was configured as a sliding wall moving at a speed of 0.3 m s^−1^, which produced shear stress. Given that the active agent in the actual experiment, sodium dodecyl benzene sulfonate, was added to the water to achieve a uniform distribution of particles, we made minor adjustments to the simulation’s water’s density and viscosity, setting them at 998.2 kg·m^−3^ and 0.001001 kg (m·s)^−1^, respectively. When the residual number dropped below 10^−4^, the calculations were deemed to have converged and allowed the flow to sufficiently develop.

### Experimental setup and data acquisition for flow measurement

In the square lid-driven cavity flow experiment, a 100 × 100 × 100 mm^3^ square chamber was utilized, and the drive belt’s tangential motion above the square chamber created the flow. The motor can be adjusted to change the belt’s motion speed. We added a slightly bigger reservoir to the upper section of the square chamber to guarantee a steady level of water in the space. To maintain a continuous experimental condition, this reservoir allowed the conveyor belt to stay submerged throughout the whole experiment. White polyethylene microspheres with a diameter of 200 µm and a density of 1 g·cc^−1^ were seeded in the water. These particles had a density of 0.072 PPM, evenly distributed throughout the flow. A massive primary vortex was created in the square cavity’s center throughout the experiment, and a secondary vortex (DSE) was created downstream of the corner zone, which was the point where the downstream sidewall and the bottom sidewall intersected. We used a light-field camera to acquire data at 40 frames per second of the secondary vortex in the DSE region. In order to showcase the RTU-Net’s efficacy in 3D imaging, we captured an image of a vortex produced by a revolving disk. The cavity flow driven by a square lid was less intricate in its axial flow structure than the vortex indicated. The disk rotation speed was set to 120 revolutions per minute.

## Supplementary information

Supplemental material

Supplementary Video 4

Supplementary Video 5

Supplementary Video 1

Supplementary Video 2

Supplementary Video 3

## Data Availability

Supporting data for RTU-Net have been made publicly available on GitHub (https://github.com/linbingzhi/RTU-Net).
