# Supplementary material for: Real-time and universal network for volumetric imaging from microscale to macroscale at high resolution
Source: Light Sci Appl. 2025 Apr 29;14:178. doi: 10.1038/s41377-025-01842-w (PMC12041240; doi:10.1038/s41377-025-01842-w)
Supplement: Supplementary file 1 — Supplemental material [file 41377_2025_1842_MOESM1_ESM.docx]

**Real-time and universal network for volumetric imaging from microscale to macroscale at high resolution**

Bingzhi Lin^1^, Feng Xing^1^, Liwei Su^1^, Kekuan Wang^1^, Yulan Liu^1^, Diming Zhang^3^, Xusan Yang^4^, Huijun Tan^1^*, Zhijing Zhu^2^*, and Depeng Wang^1^*

^1^College of Energy and Power Engineering, Nanjing University of Aeronautics and Astronautics, Nanjing, 210016, China

^2^Key Laboratory of Novel Targets and Drug Study for Neural Repair of Zhejiang Province, School of Medicine, Hangzhou City University, Hangzhou, 310015, China

^3^Key Laboratory of Soybean Molecular Design Breeding, National Key Laboratory of Black Soils Conservation and Utilization, Northeast Institute of Geography and Agroecology, Chinese Academy of Sciences, Changchun 130102, China.

^4^Institute of Physics Chinese Academy of Sciences, Beijing, 100190, China

[*tanhuijun@nuaa.edu.cn](mailto:*tanhuijun@nuaa.edu.cn)

*vczzj@zju.edu.cn

[*depeng.wang@nuaa.edu.cn](mailto:*depeng.wang@nuaa.edu.cn)

**
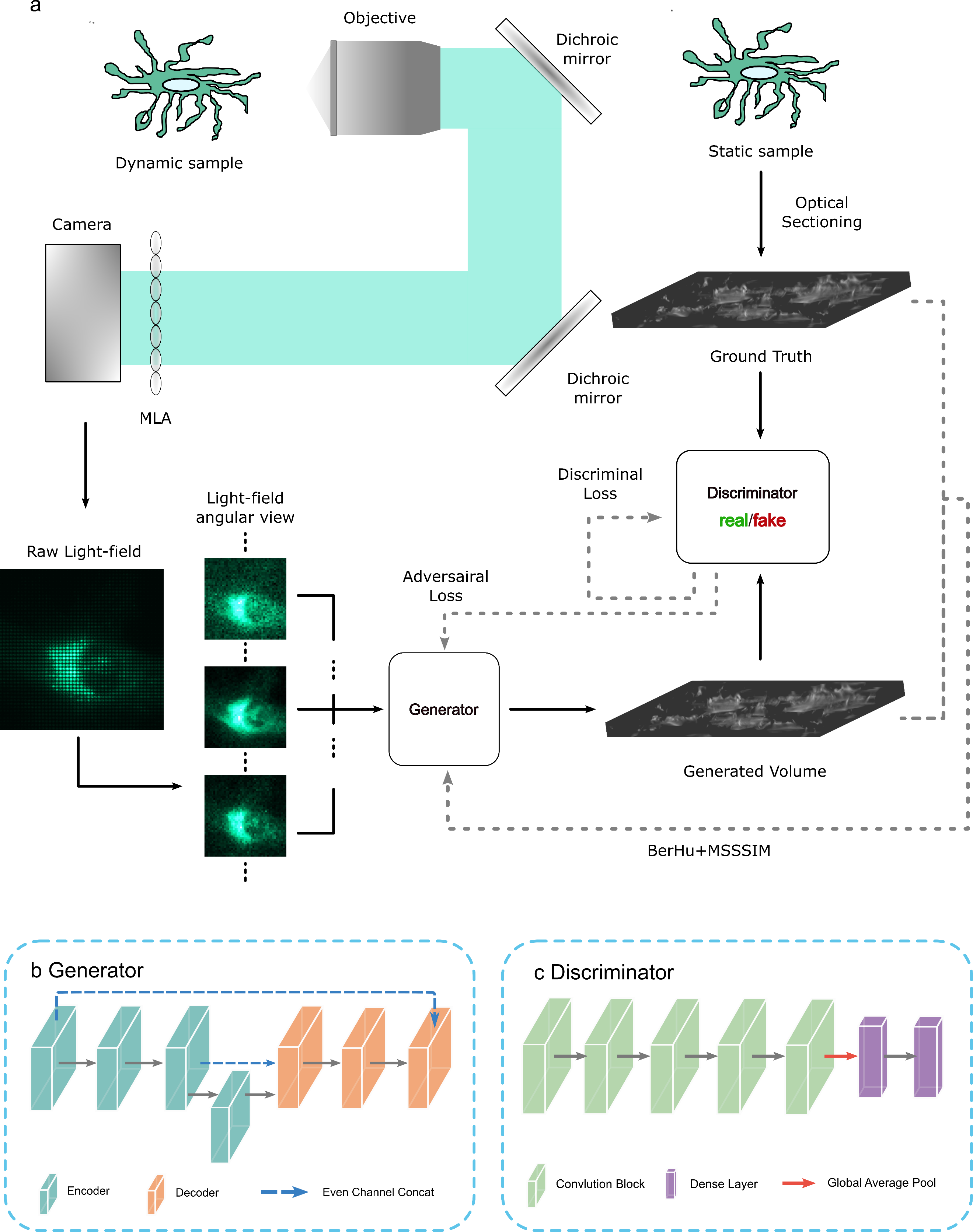
**

**Fig. S1 | Experiment setup and** **detailed network structure of RTU-Net.** **a,** The GAN structure and the data flow of RTU-Net. **b,** The generator structure of RTU-Net. **c,** The discriminator structure was used to train RTU-Net.

**
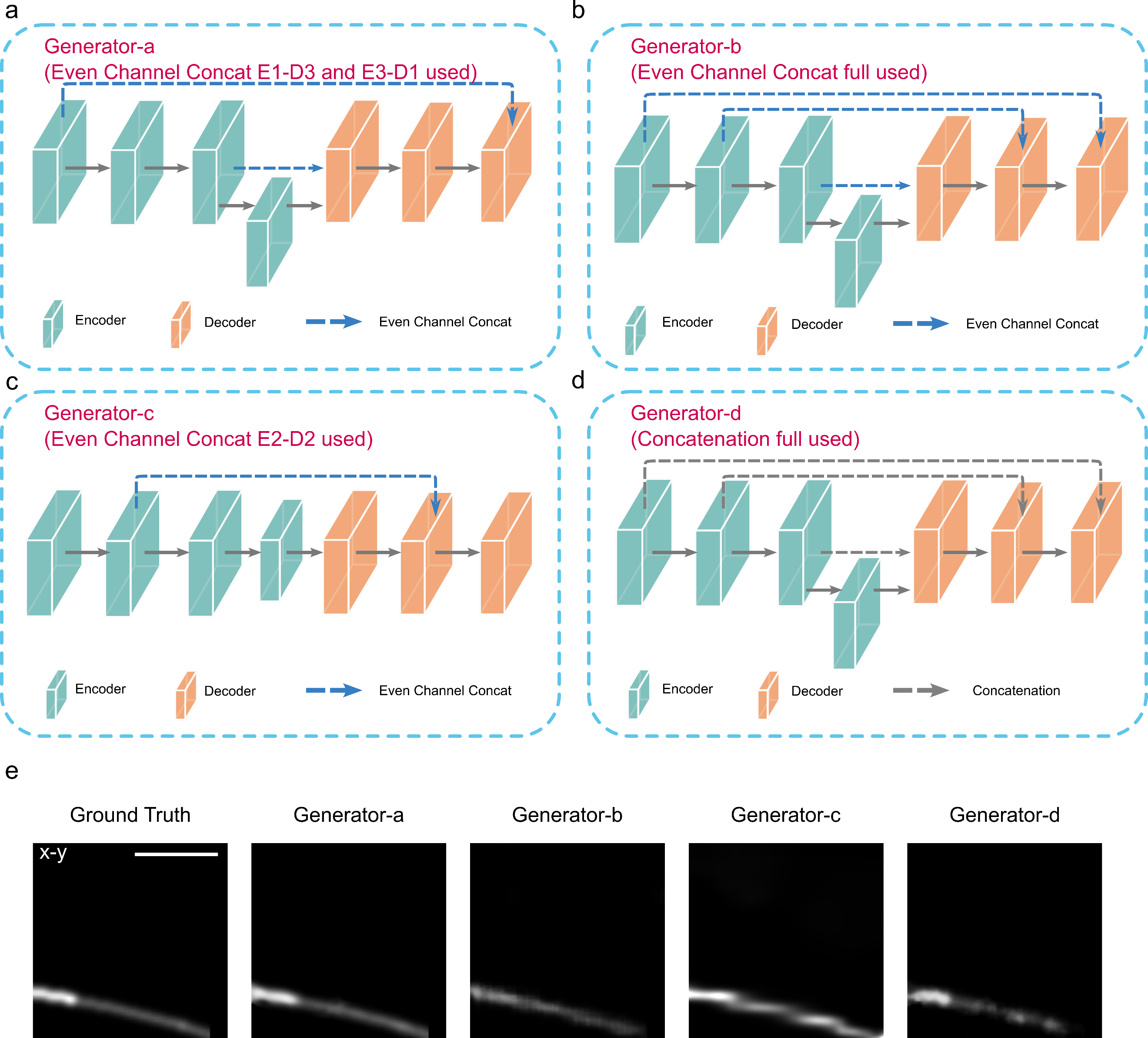
**

**Fig. S2 | Ablation study on RTU-Net (Generator).** **a,** Simplified schematic of Generator-a, with Even Channel Concat E1-D3 and E3-D1, used. **b,** Simplified schematic of Generator-b, with Even Channel Concat fully used. **c,** Simplified schematic of Generator-c, with Even Channel Concat E2-D2 used. **d,** Simplified schematic of Generator-d, with Concatenation fully used. **e,** The simulated ground truth(left), corresponding output results obtained by Generator-a, Generator-b, Generator-c, and Generator-d. Scale bar, 50 μm

**
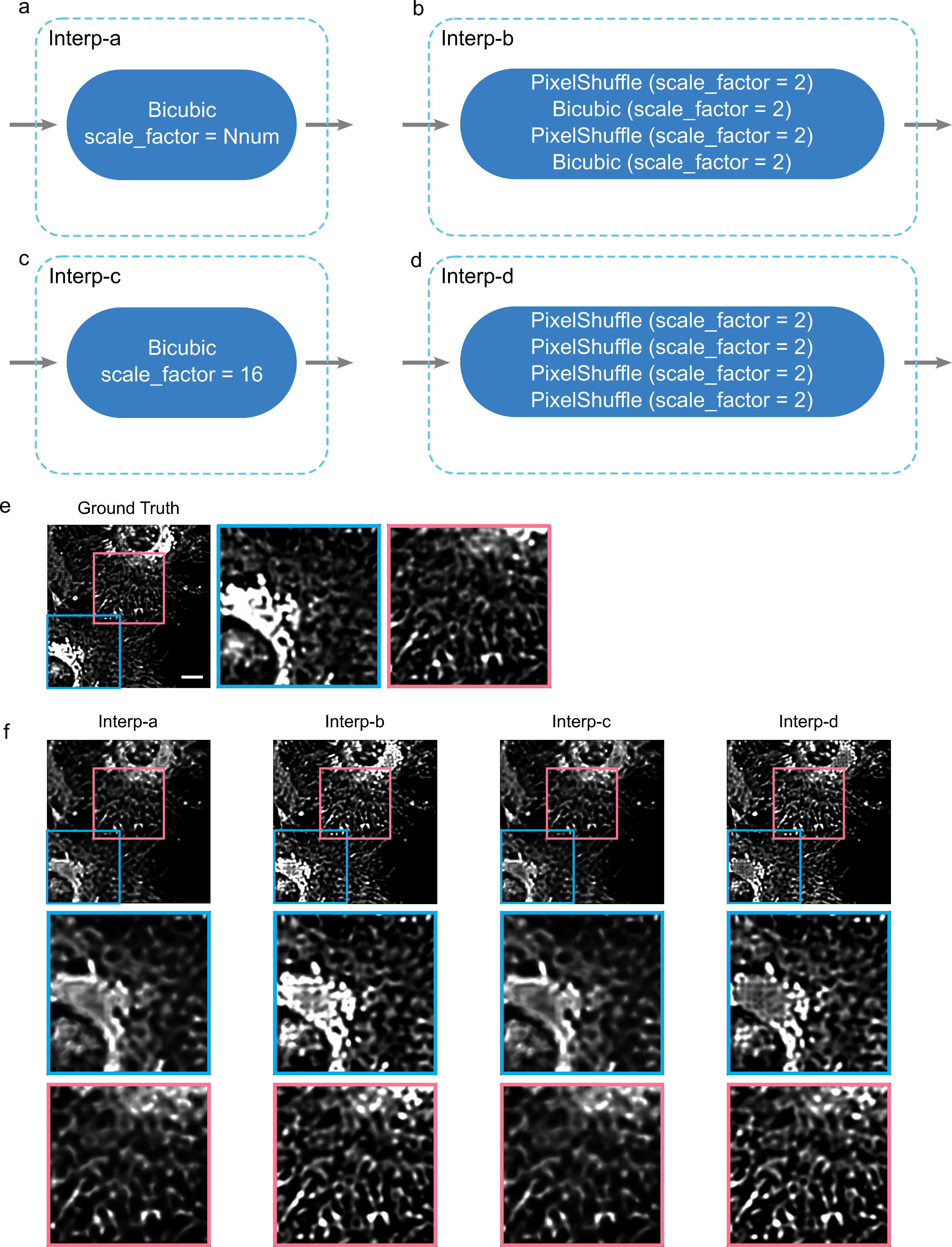
**

**Fig. S3 | Ablation study on RTU-Net-interpolation part. a,** Simplified schematic of Interp-a. It uses bicubic upsampling, and the scale_factor used is Nnum. **b,** Simplified schematic of Interp-b with alternative pixelshuffle and bicubic is used, and the scale factor is 2. **c,** Simplified schematic of Interp-a. It uses bicubic upsampling, and the scale_factor used is 16. **d,** Simplified schematic of Interp-b, with only the PixelShuffle used, and the scale factor is 2. **e,** Orthogonal MIPs of a fixed L929 cell with membrane, regarded as ground truth. **f,** Corresponding output results obtained by Interp-a, Interp-b, Interp-c, and Interp-d. Scale bar, 5 μm**.**

**
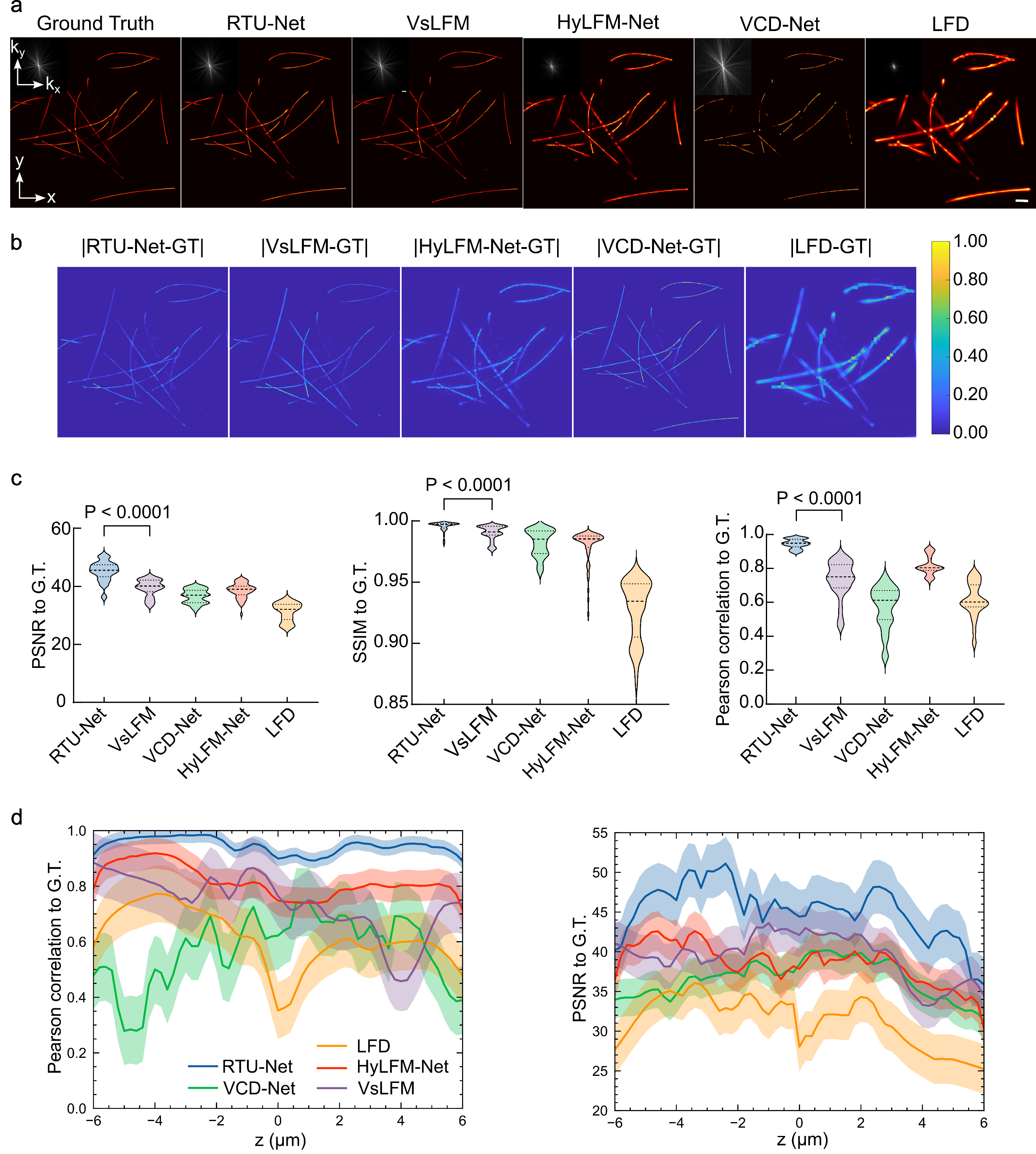
**

**Fig. S4 | The performance of RTU-Net on synthetic 1-μm-diameter tubulins**. **a,** Orthogonal MIPs of 1-μm-diameter synthetic tubulins, acquired by sLFM with a ×63/1.4 NA oil-immersion objective in ideal imaging conditions, regarded as ground truth, MIPs of obtained by RTU-Net, VsLFM, HyLFM-Net, VCD-Net, and LFD trained on the same type of sample and The corresponding Fourier spectrum. **b,** Compare the corresponding difference maps of RTU-Net, VsLFM, HyLFM-Net, VCD-Net, and LFD with GT. **c,** Violin plots showing PSNR, SSIM, and Pearson correlation of results obtained by RTU-Net, VCD-Net, HyLFM-Net, VsLFM, and LFD, compared with ground truth. The center line represents the median, the box limits represent the lower and upper quartiles, and the whiskers represent 1.5-fold the interquartile range. n = 61 for each method. P values were calculated using the two-sided paired t-test: P <0.0001 for VsLFM. **d,** Quantitatively compared the reconstruction fidelity of RTU-Net, VsLFM, HyLFM-NET, VCD-Net, and LFD via layer-by-layer calculating their Pearson correlation and PSNR indices, using GT as reference. Scale bar: 5 μm.


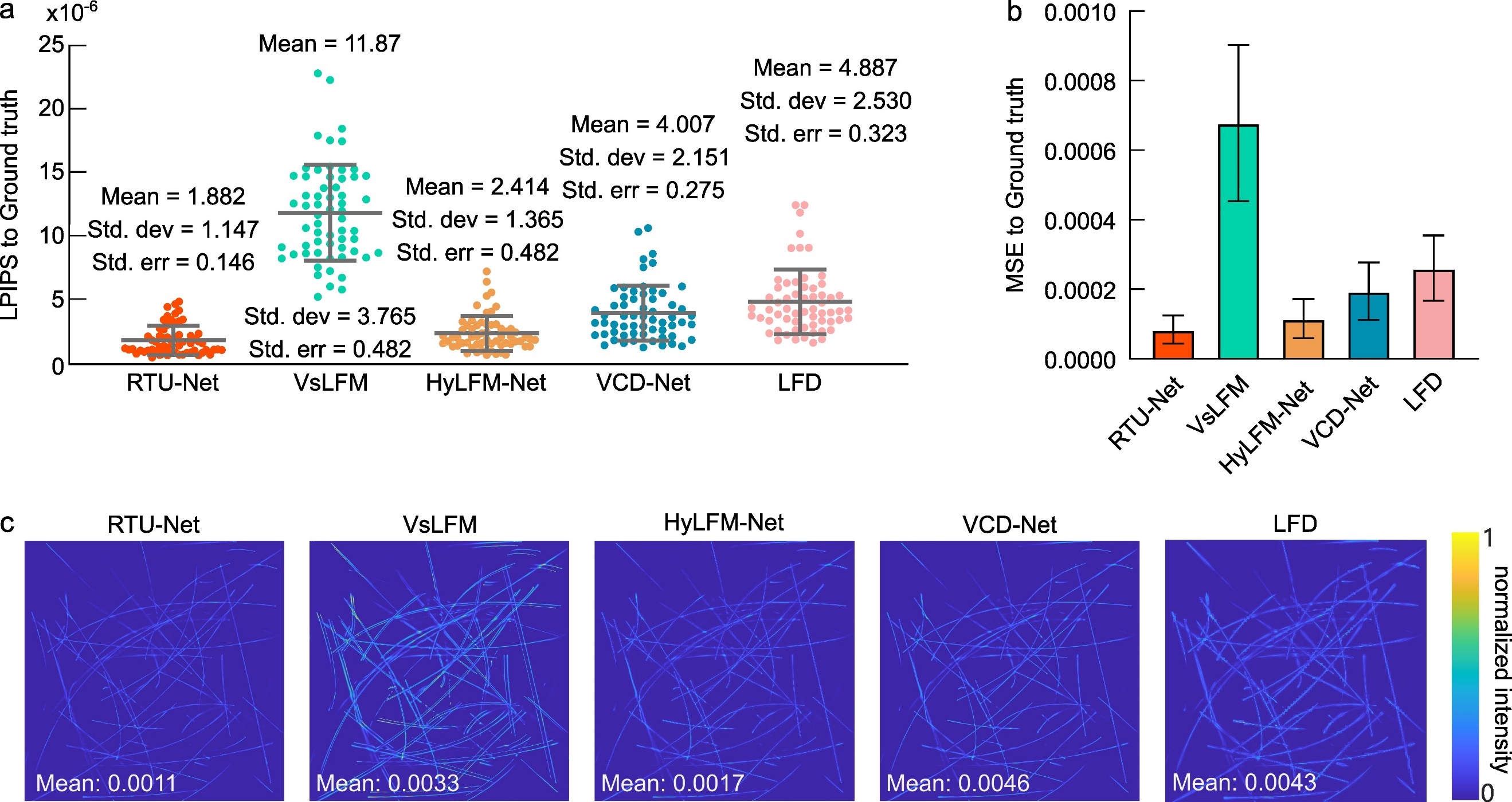


**Fig. S5 | Comparison results for 1 μm tubulins with other metrics.** **a,** LPIPS of results obtained by RTU-Net, VsLFM, HyLFM-Net VCD-Net, and LFD, compared with ground truth. The center line represents the median, the box limits represent the lower and upper quartiles, and the whiskers represent 1.5-fold the interquartile range, n =61, mean±std. **b,** MSE bar chart from RTU-Net, VsLFM, HyLFM-Net VCD-Net, and LFD， compared with ground truth. n =61, mean±std. **c,** Compare RTU-Net, VsLFM, HyLFM-Net VCD-Net, and LFD with ground truth in the corresponding difference maps.

**
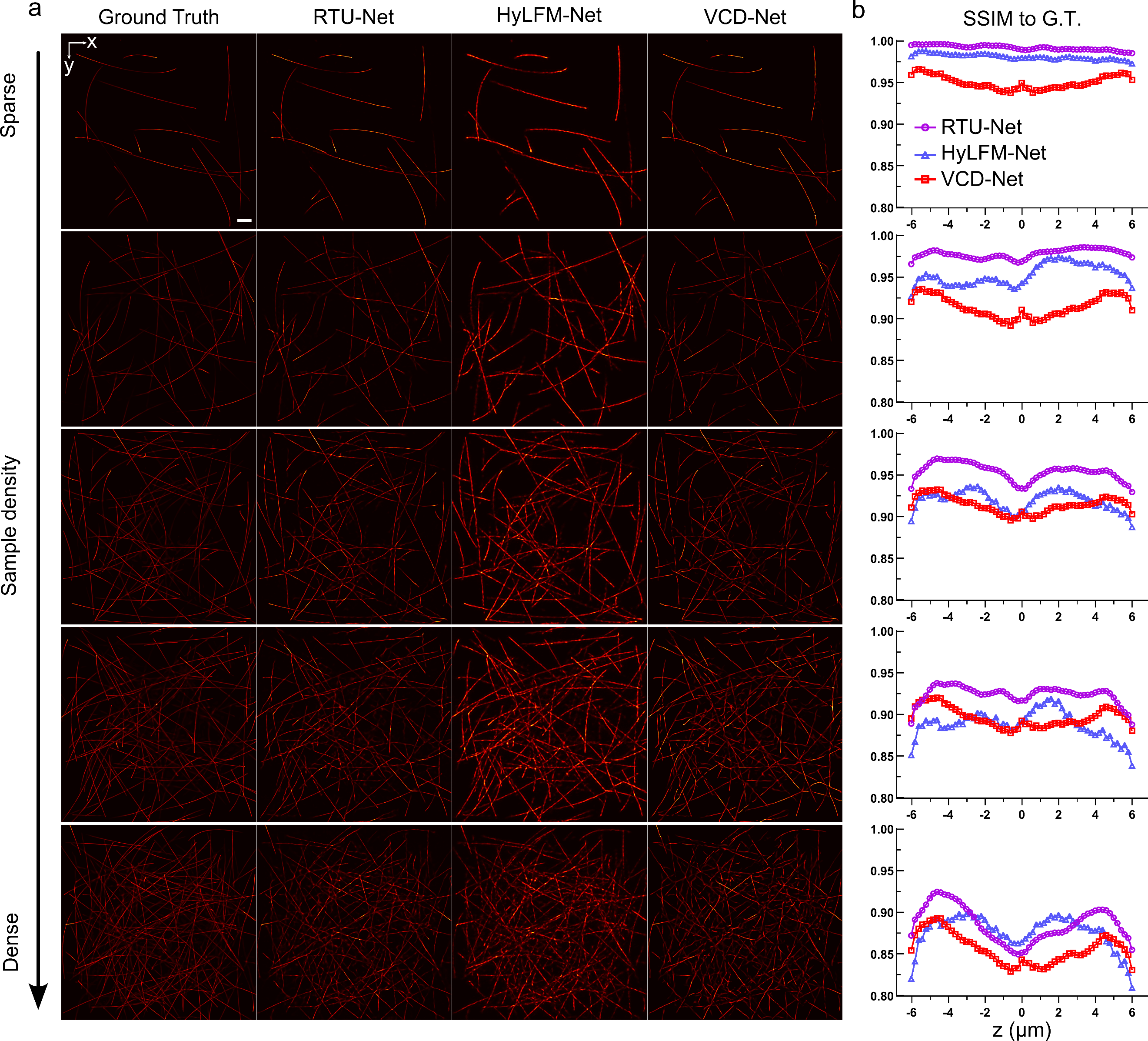
**

**Fig. S6 | Evaluation of RTU-Net imaging performance on synthetic tubulins at different sample densities. a,** x-y-MIPs stitched by RTU-Net, HyLFM-Net, and VCD-Net results, with gradually increasing sample densities. **b,** The curves show the five density SSIM obtained by RTU-Net, HyLFM-Net, and VCD-Net in comparison to the ground truth. Scale bar, 50 μm.

**
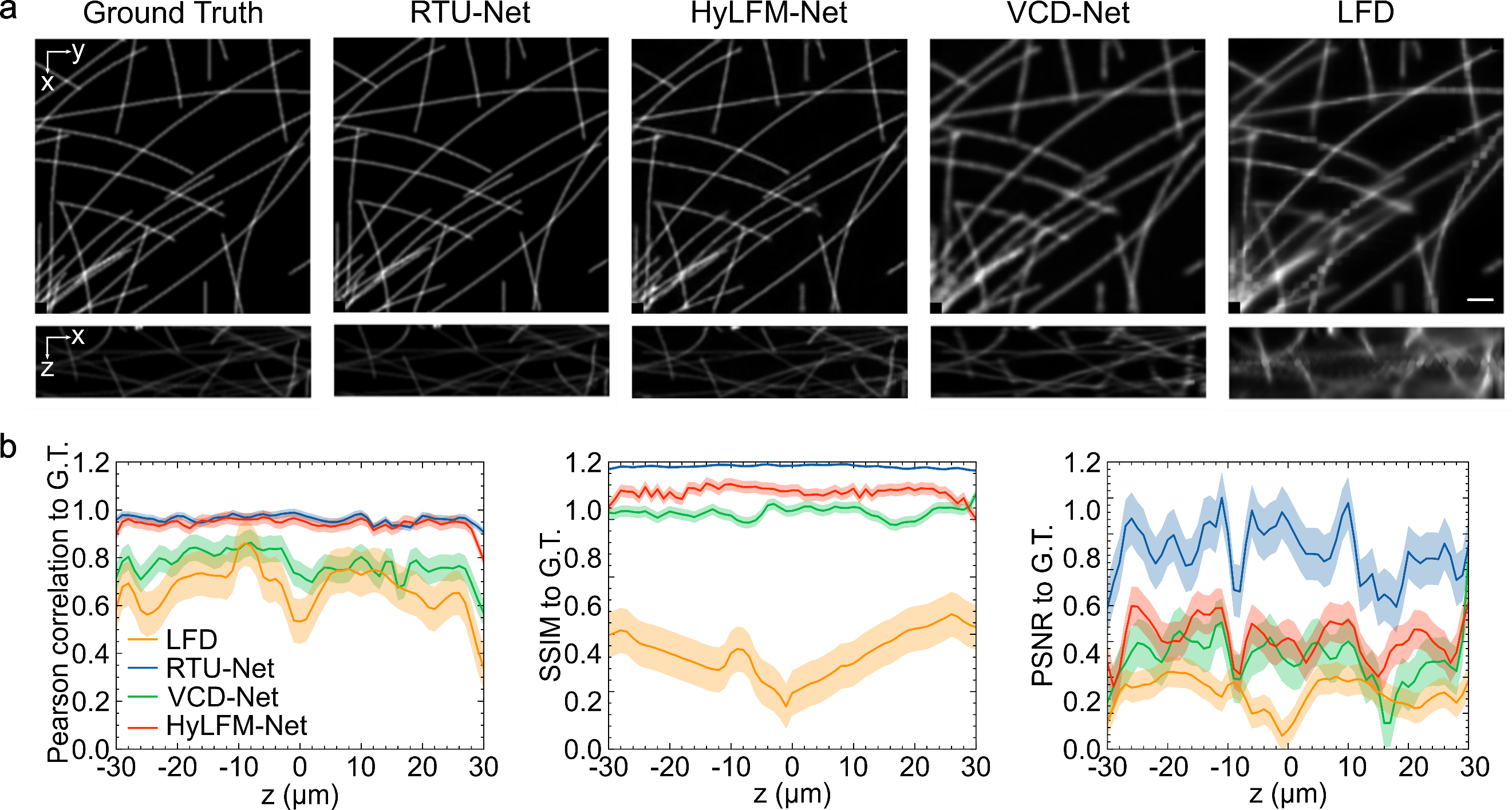
**

**Fig. S7 | The performance of RTU-NET trained on 10-μm-diameter synthetic tubulins. a,** Maximum intensity projections (MIPs) of the same tubulins, obtained by RTU-Net, HyLFM-Net, VCD-Net, and LFD, respectively. **b,** Quantitative reconstruction fidelity comparison of RTU-Net, HyLFM-NET, VCD-Net, and LFD via layer-by-layer calculating their Pearson correlation, SSIM, and PSNR indices, using GT as reference. Scale bars, 50 μm.

**
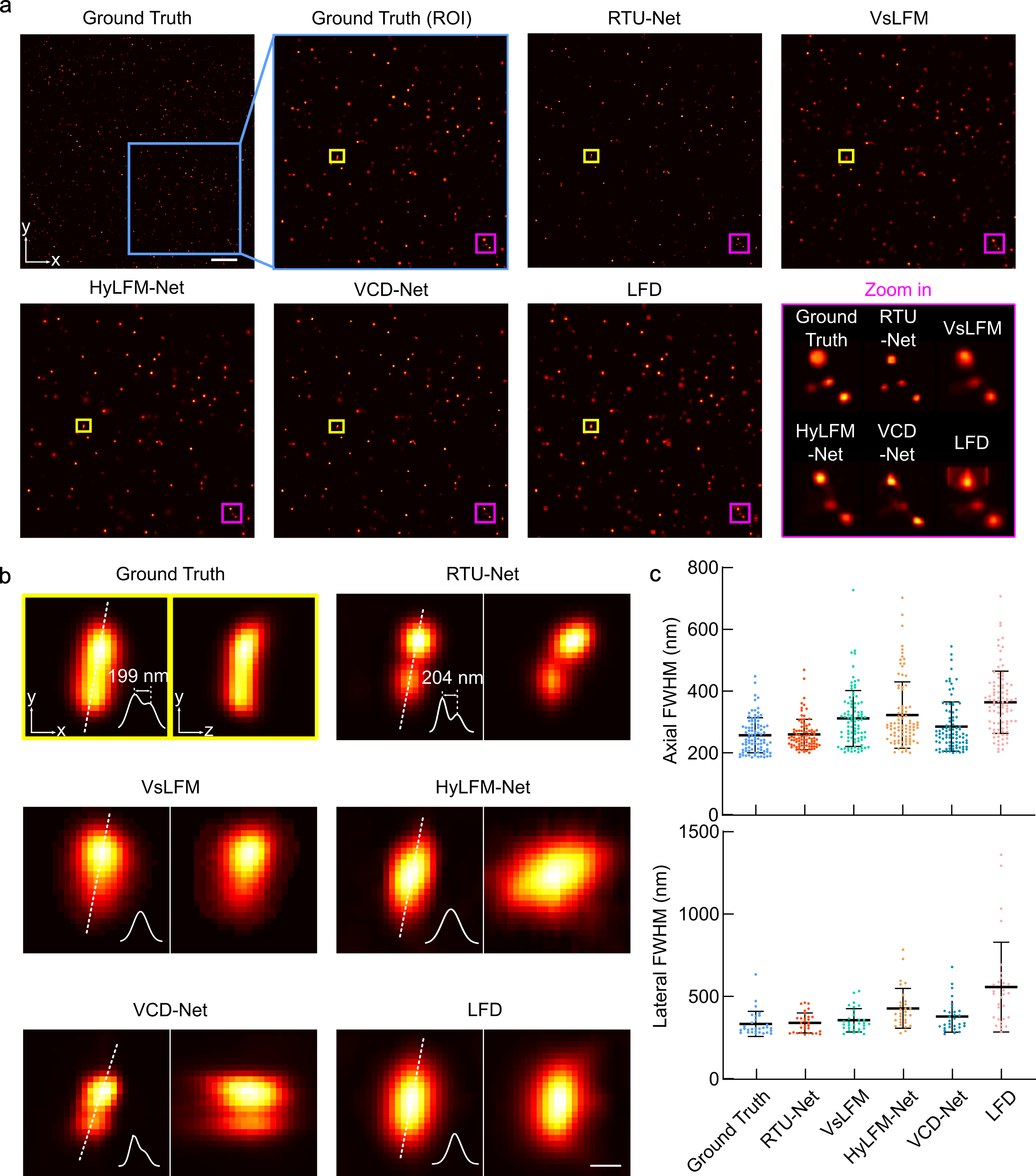
**

**Fig. S8 | RTU-Net performance on 100-nm-diameter fluorescent beads. a,** The MIP area of x-y was imaged using sLFM (ground truth). It consists of 100-nm diameter fluorescent beads that are uniformly distributed in low-melting agarose. The MIP blue domain was obtained separately by RTU-Net, VsLFM, HyLFM-NET, VCD-NET, and LFD. The zoom-in is an enlarged purple area. **b,** Enlarged the MIP yellow region in (**a**), and measured the FWHM with a Gaussian fit. **c,** Boxplots of averaged axial full width at half maximum resolution of Ground truth, RTU-Net, VsLFM, HyLFM-Net, VCD-Net, and LFD at x-y MIPs (n=50). Scale bars, 10 μm **(a)**, 500 nm **(e).**

**
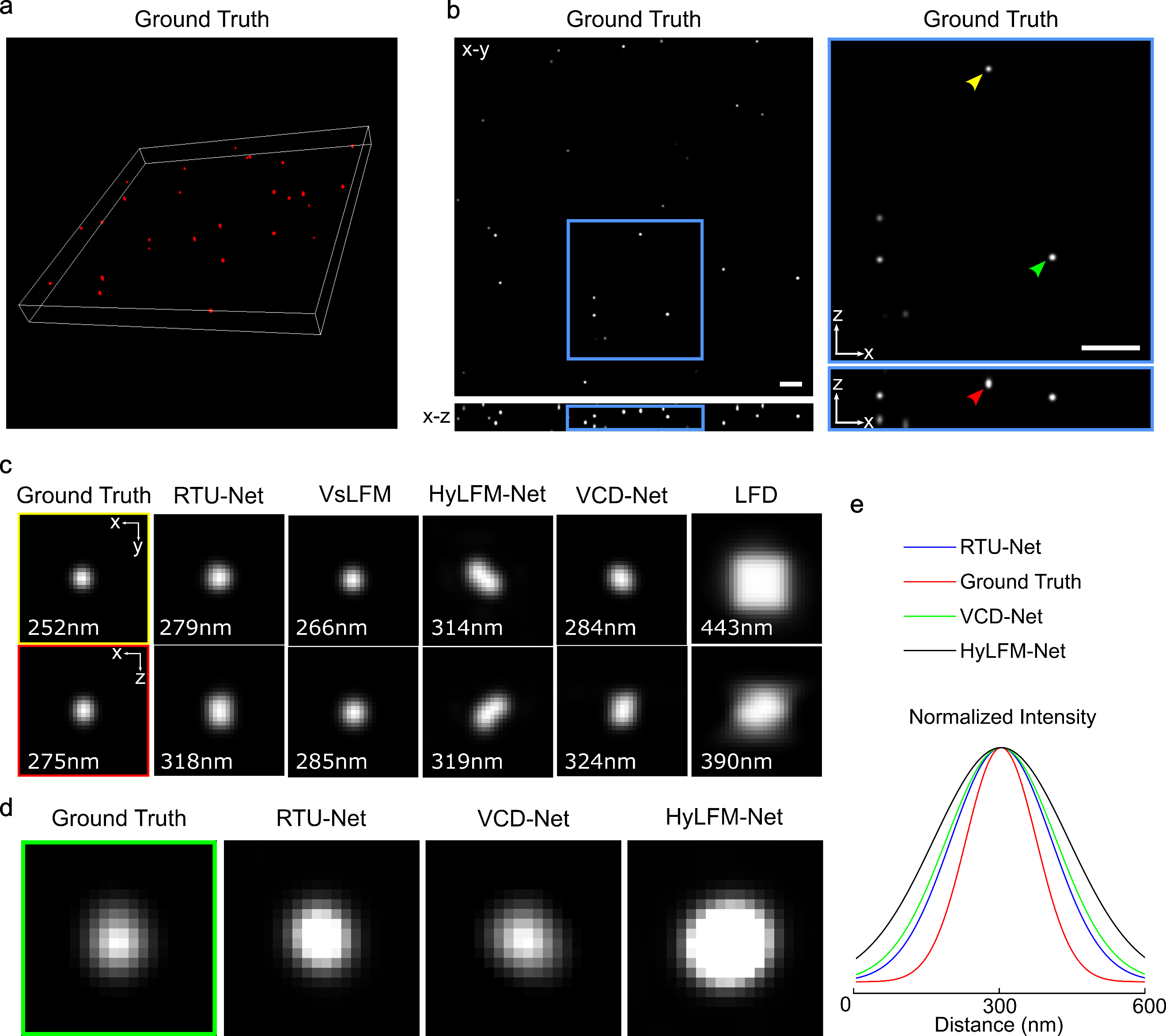
**

**Fig. S9 | Comparisons of resolution at different axial positions among LFM, VCD-Net, HyLFM-Net, VsLFM, and sLFM. a,** Microbeads three-dimensionally visualized using sLFM (GT) results. **b,** Whole-FOV and enlarged MIPs of 100-nm fluorescence beads. **c,** Enlarged MIPs of individual reconstructed beads and FWHMs at different methods (RTU-Net, VsLFM, HyLFM-Net, VCD-Net, and LFD). The selected beads are marked by yellow arrow and red arrow. **d,** Enlarged MIPs of individual reconstructed beads at different methods (RTU-Net, HyLFM-Net, and VCD-Net). The selected beads are marked by green arrow. **e,** The axial intensity profiles for selected beads are marked by green arrow. Scale bar, 5 µm.

**
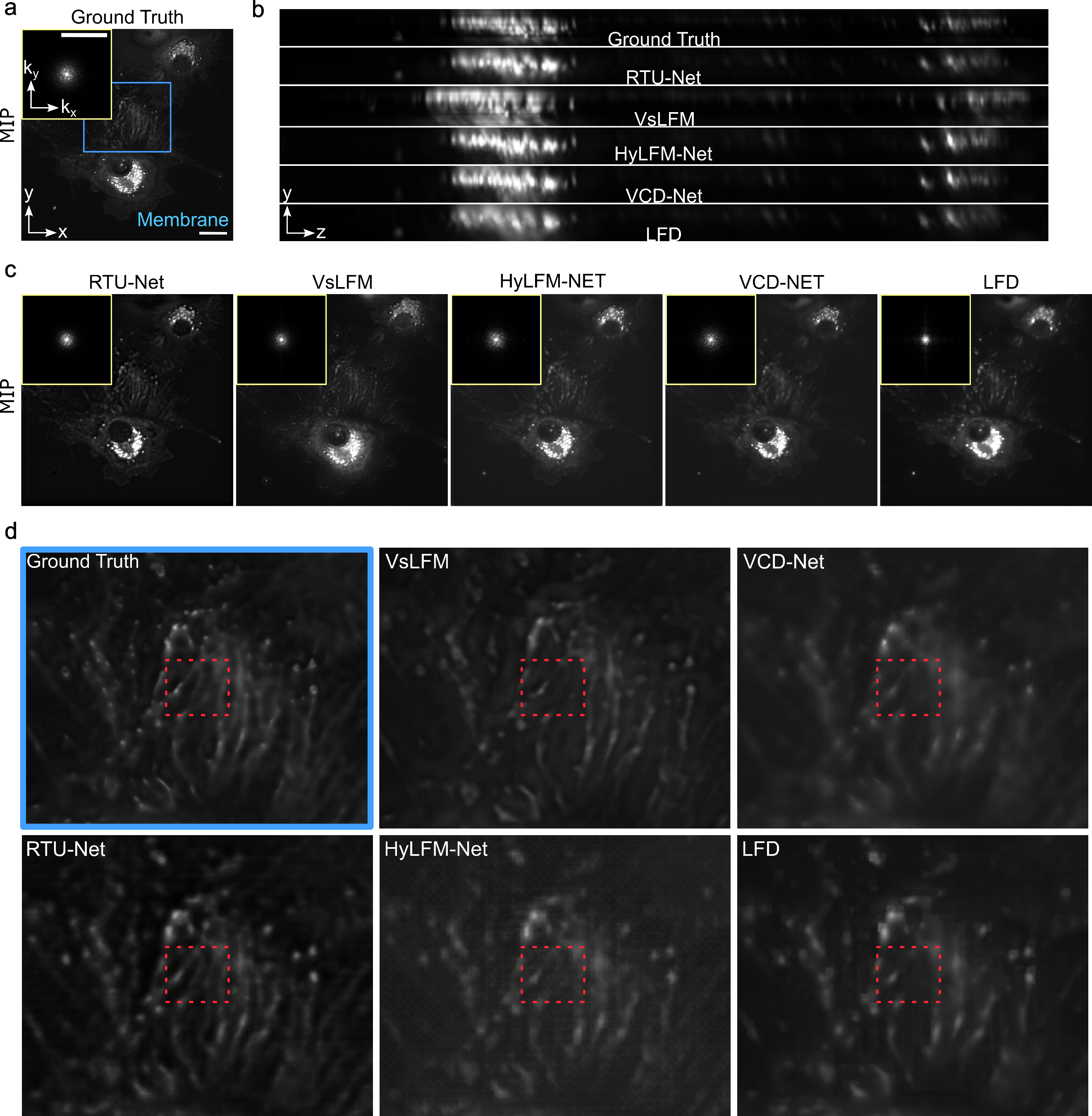
**

**Fig. S10 | Reconstruction volumes comparison between RTU-Net and other algorithms on membrane imaging. a,** The Maximum Intensity Projection (MIP) of the membrane and the Fourier spectrum were both plotted onto the MIP images. **b,** The MIP area of y-z was imaged Ground Truth, RTU-Net, VsLFM, HyLFM-Net, VCD-Net, and LFD. **c,** MIPs of a fixed L929 cell with membrane, obtained by RTU-Net, VsLFM, HyLFM-Net, VCD-Net, and LFD trained on the same type of sample (membrane). The corresponding Fourier spectrum were both plotted onto the MIP images. **d,** Enlarged MIPs of reconstructed volumes using different methods (RTU-Net, VsLFM, HyLFM-Net, VCD-Net, and LFD). The selected area is marked by **(a)** blue region.

**
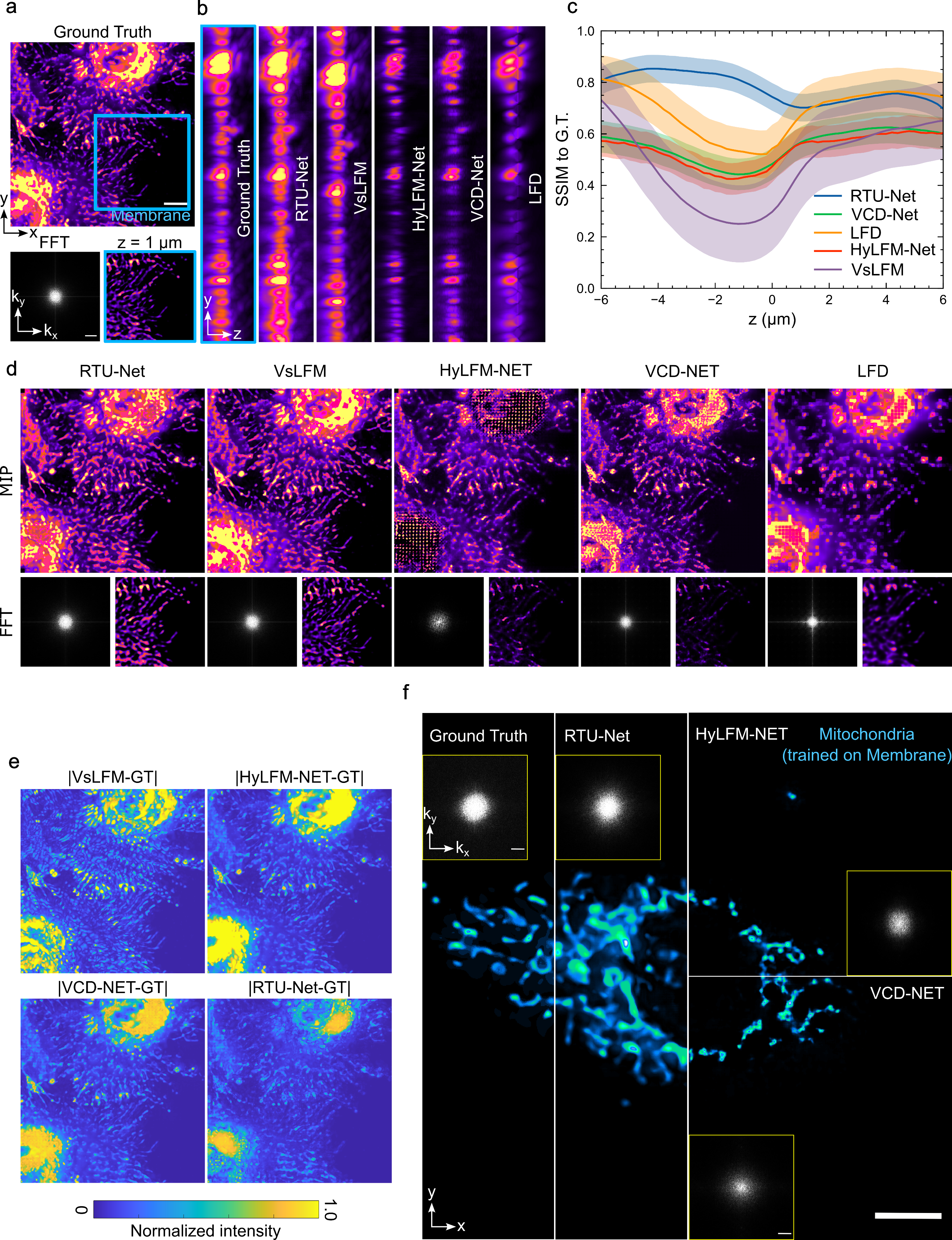
**

**Fig. S11 | Generalization ability of RTU-Net trained on membrane data. a,** Orthogonal MIPs of a fixed L929 cell with membrane, acquired by sLFM with a ×63/1.4 NA oil-immersion objective in ideal imaging conditions, regarded as ground truth, Fourier spectrum, and enlarged blue region are shown in the bottom row. **b,** The MIP area of y-z was imaged Ground Truth, RTU-Net, HyLFM-Net, VCD-Net, VsLFM, and LFD**. c,** SSIM of membrane obtained by RTU-Net, HyLFM-Net, VCD-Net, VsLFM, and LFD compared with ground truth. **d,** MIPs of a fixed L929 cell with membrane, obtained by RTU-Net, VsLFM, HyLFM-Net, VCD-Net, and LFD trained on the same type of sample (membrane). The corresponding Fourier spectrum and enlarged blue region are shown at the bottom of each panel. **e,** The MIPs of mean absolute errors (MAE) obtained by VCD-Net, HyLFM-Net, VsLFM, and RTU-Net were calculated layer-by-layer on the same type of membrane. **f,** MIPs of a fixed L929 cell with mitochondria, obtained by RTU-Net, HyLFM-Net, and VCD-Net trained on the same type of membrane. The corresponding Fourier spectrum is shown at the bottom of each panel. Scale bar, 5 μm.


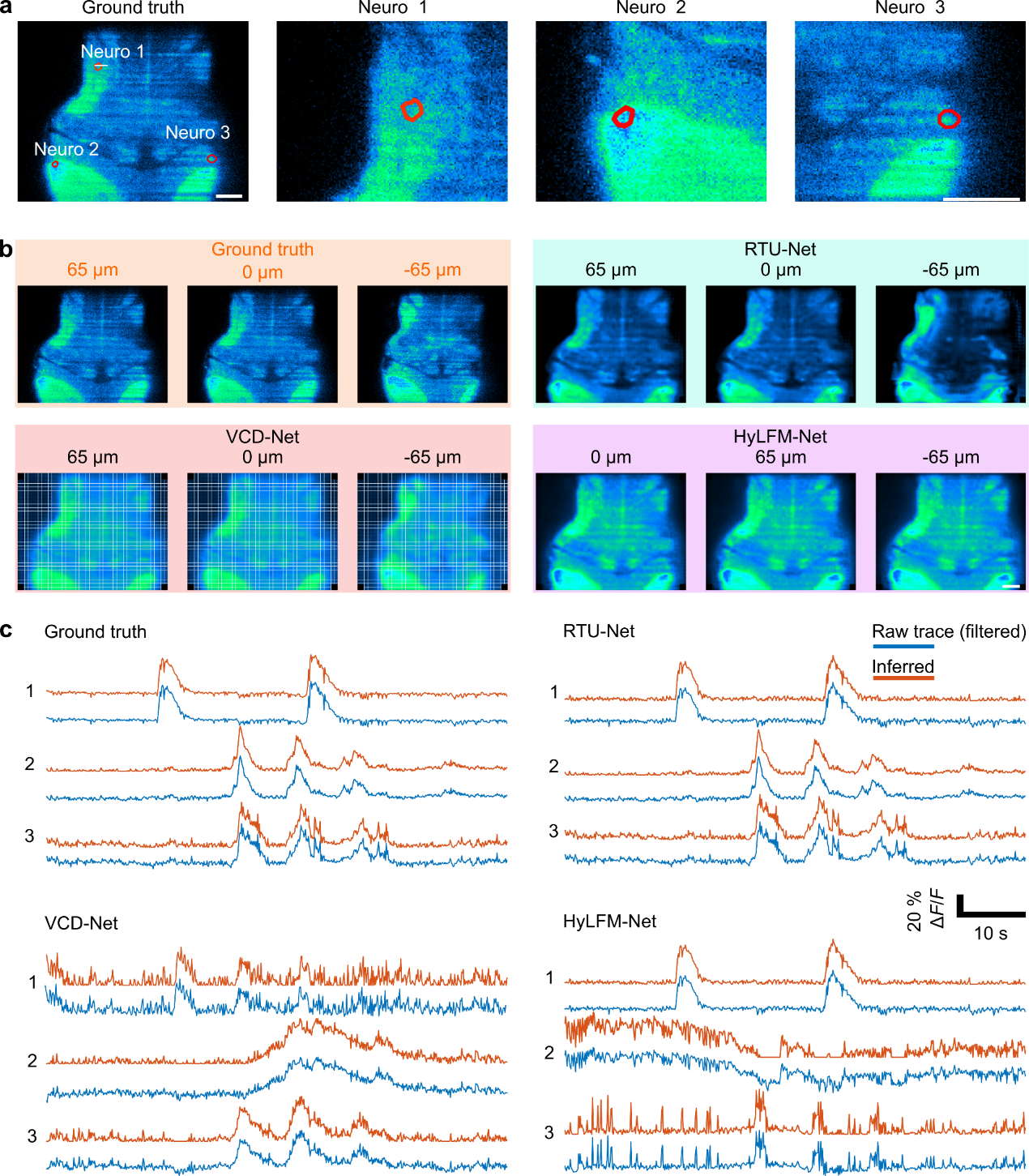


**Fig. S12 | RTU-Net performance on light field imaging of alive zebra fish larvae brain expressing fluorescence protein.** **a,** The ground truth of zebrafish brain acquired with light sheet microscopy. The enlarged views show the selected neuro section. **b,** The comparison of ground truth and the images reconstructed by RTU-Net, VCD-Net and HyLFM-Net. Representative images of the ground truth zebrafish brain, and the brain images reconstructed by RTU-Net, VCD-Net and HyLFM-Net. **c,** The neural activity extracted from the ground truth volumes, and the volumes reconstructed by RTU-Net, VCD-Net and HyLFM-Net. Scale bar: 20 μm.

**
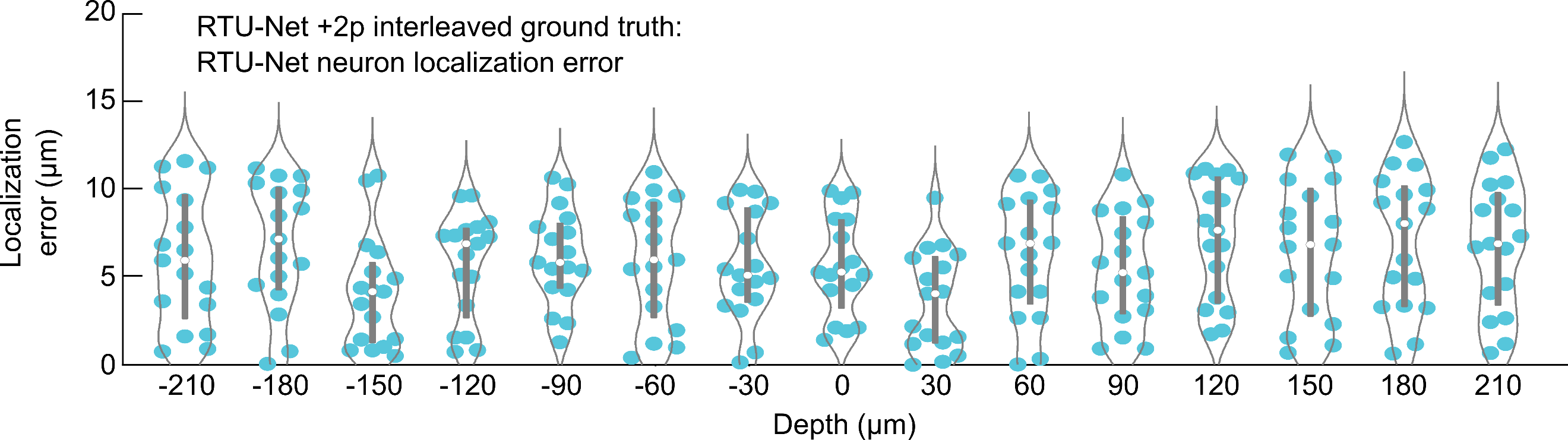
 Fig. S13 | Distributions of axial neuron localization errors between RTU-Net extracted neuron positions and ground truth.** The white circle represents the median; the thick gray vertical line shows the interquartile range; thin vertical lines indicate the upper and lower proximal values; transparent blue disks show data points; and the transparent violin-shaped area shows the data distribution, n = 17 neuron pairs.


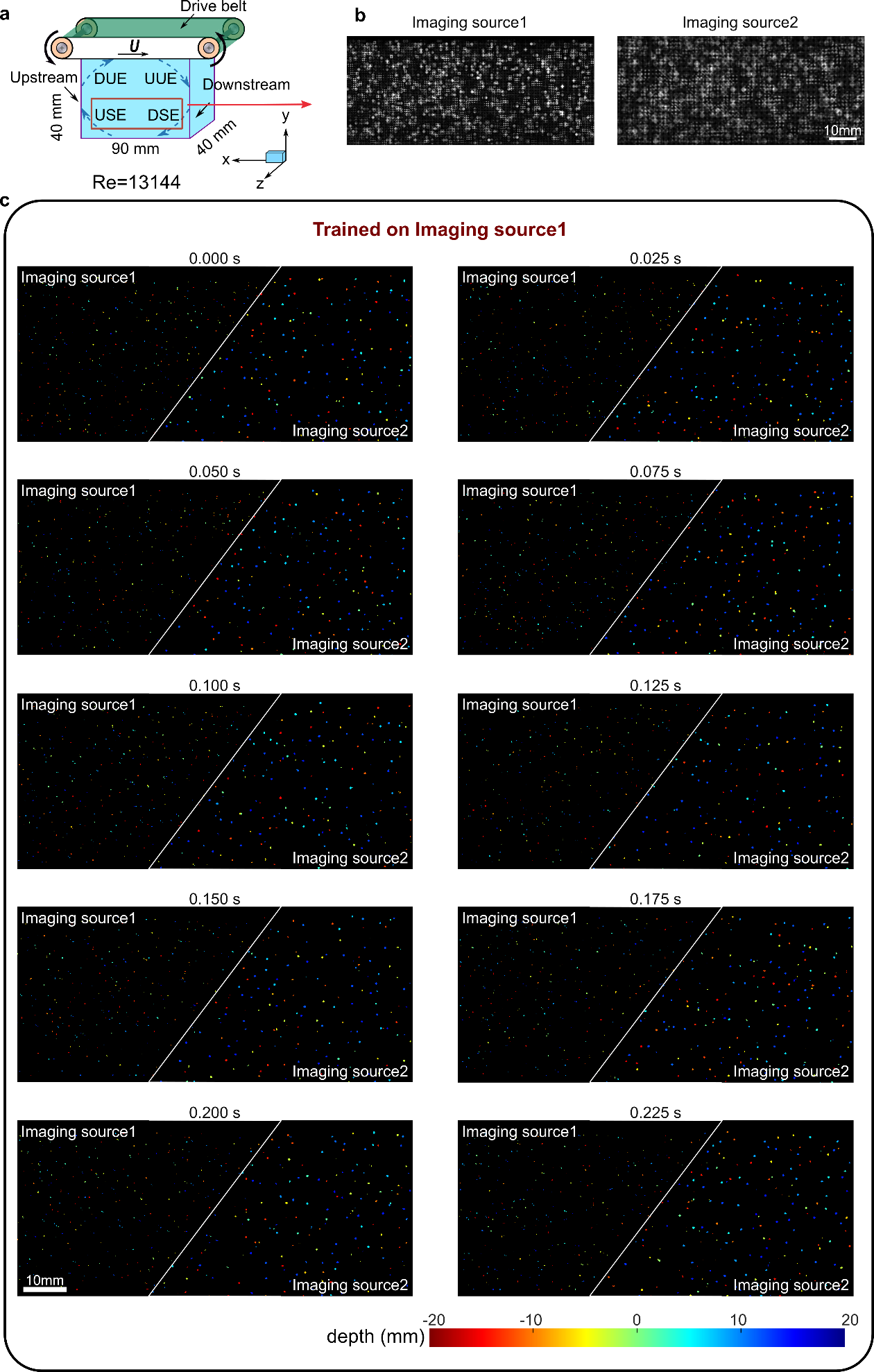


**Fig. S14 | Verification of RTU-Net's generalization capability with different imaging sources in a square-cavity-driven flow.** **a,** Schematic diagram of a square lid-driven cavity flow measurement device with the imaging area shown in the red region. **b,** Light field images of the imaging region from two different imaging sources. **c,** Particles trained on imaging source 1, predicting flow particle results for imaging source1 and imaging source2. Scale bar: 10 mm.

**
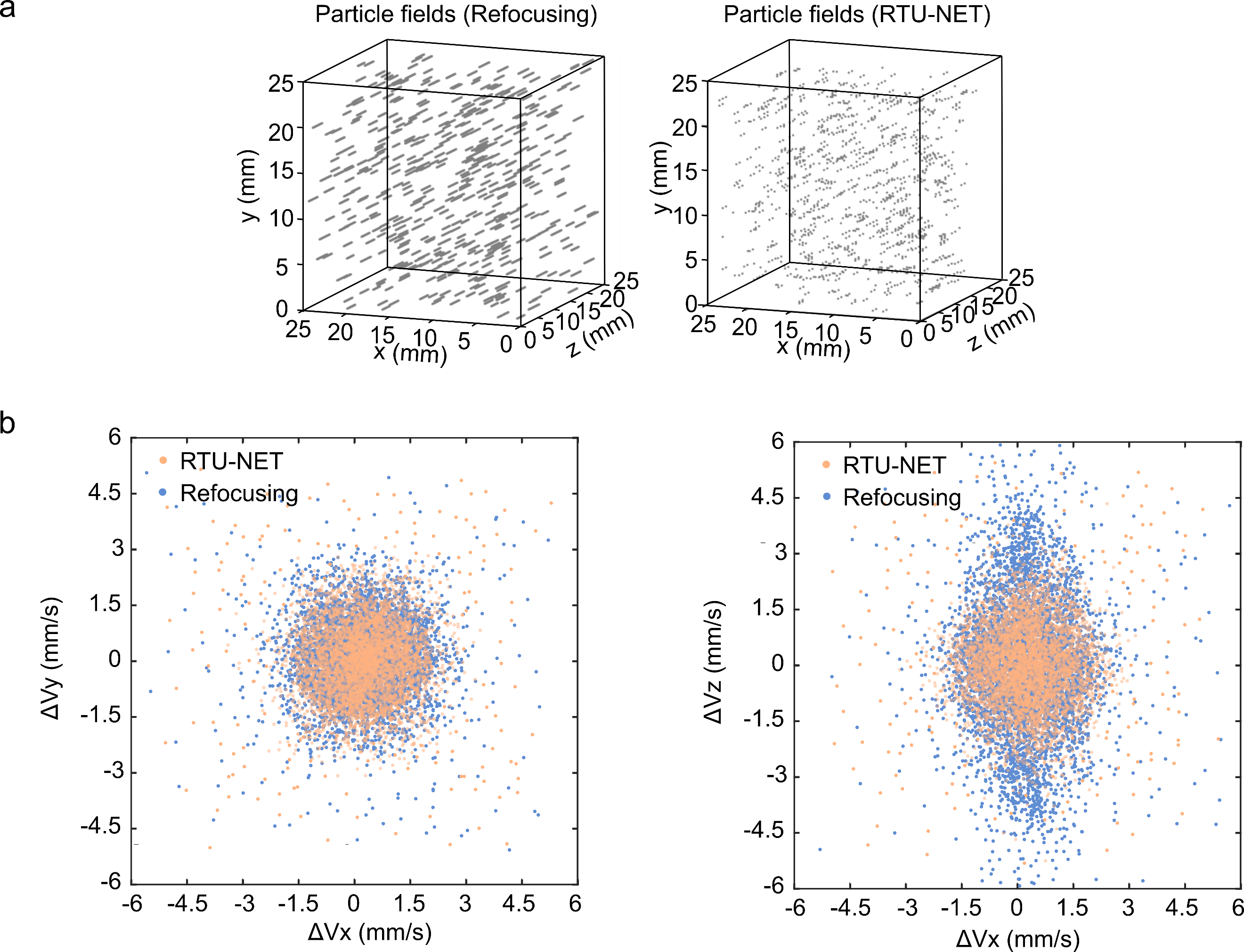
**

**Fig. S15 | Measurement of LF-PIV system by** RTU-Net. **a,** The particle fields obtained from Refocusing and RTU-Net. **b,** Obtained velocity error maps for xy and xz planes using ANSYS Fluent as the Ground truth.

**
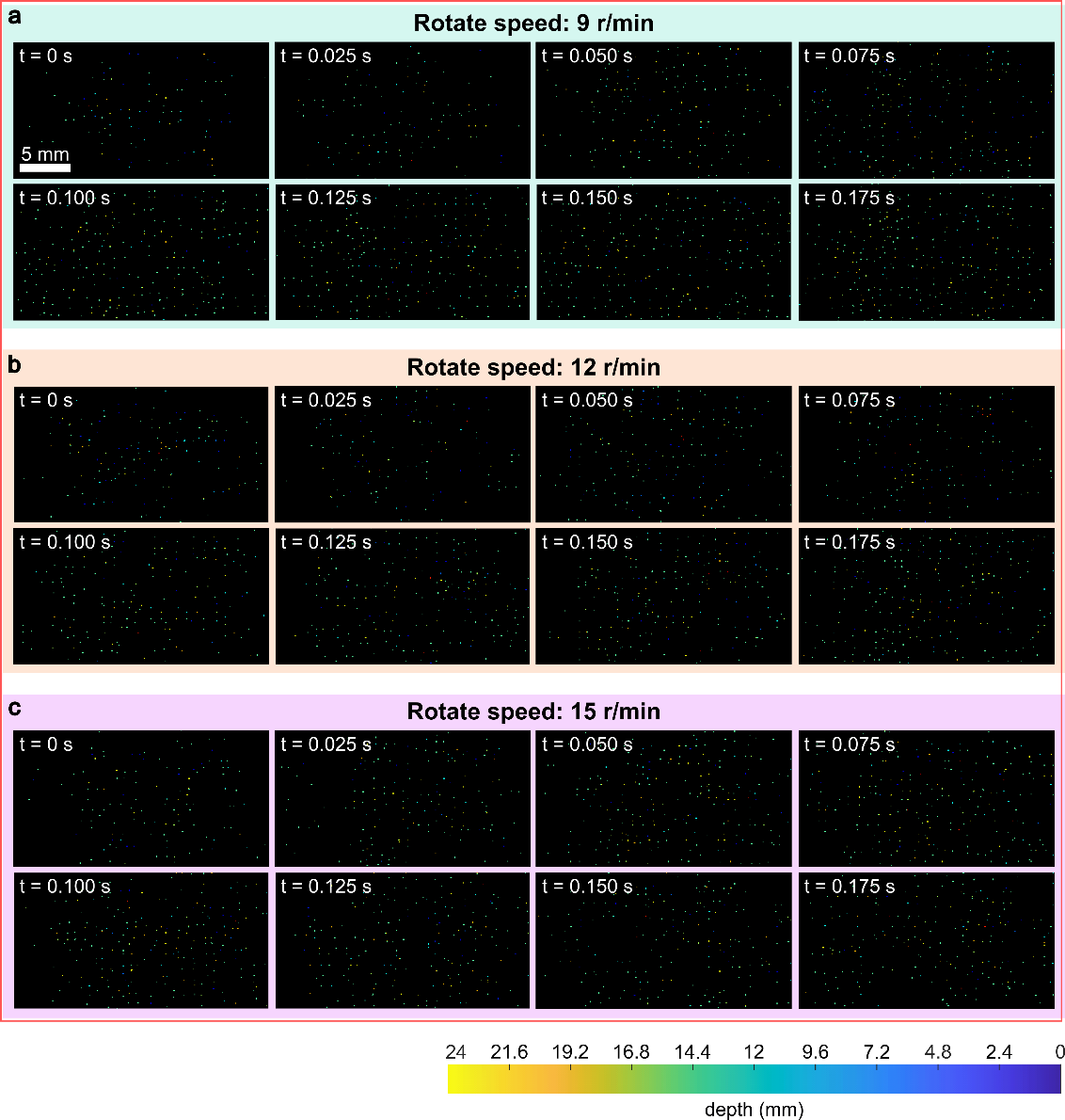
**

**Fig. S16 | Reconstruction results of vortex dynamics at different rotational speeds.** **a,** Dynamic reconstruction results for different moments at a rotor speed of 9 r·min^-1^. **b,** Dynamic reconstruction results for different moments at a rotor speed of 12 r·min^-1^. **c,** Dynamic reconstruction results for different moments at a rotor speed of 5 r·min^-1^. Scale bar: 5 mm.

**
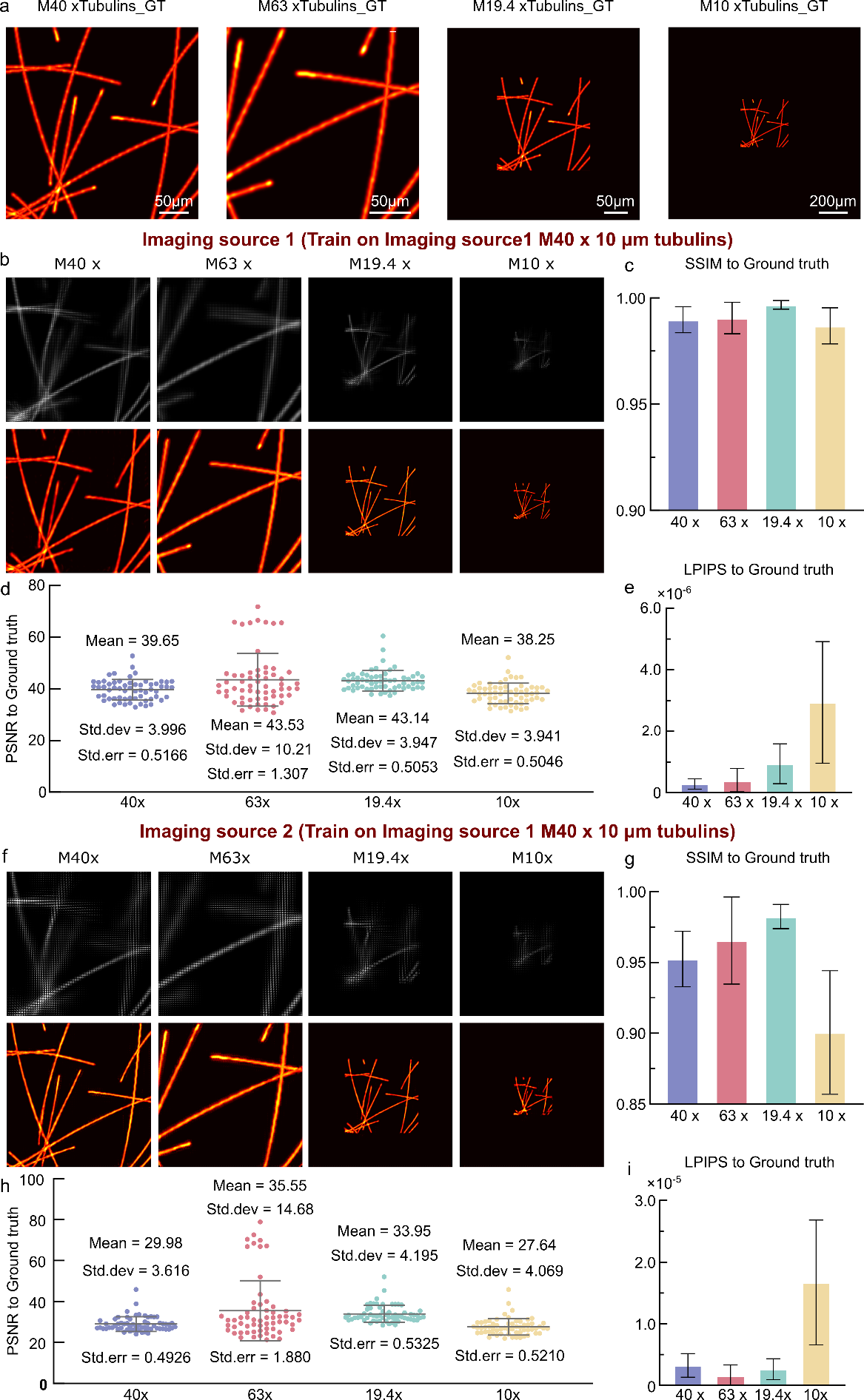
**

**Fig. S17 |** **Generalization ability between different imaging sources on 10 μm tubulins at different scales. a,** MIPs of 10 µm tubulin at different magnifications (M40×, M63×, M19.4×, M10×) were considered ground truth. **b,** Light field images at different scales under imaging source1 (upper), trained on imaging source1 M40×, and reconstruction results at different scales (bottom). **c,** Bar chart of SSIM results at M40×, M63×, M19.4×, and M10× reconstructed from imaging source1, compared with ground truth. n =61, mean±std. **d,** PSNR of results obtained by M40×, M63×, M19.4×, and M10×, compared with ground truth. The center line represents the median, the box limits represent the lower and upper quartiles, and the whiskers represent 1.5-fold the interquartile range, n =61, mean±std. **e,** Bar chart of LPIPS results at M40×, M63×, M19.4×, and M10× reconstructed from imaging source1, compared with ground truth. n =61, mean±std. **f,** Light field images at different scales under imaging source2 (upper), trained on imaging source1 M40×, and reconstruction results at different scales (bottom). **g,** Bar chart of SSIM results at M40×, M63×, M19.4×, and M10× reconstructed from imaging source2, compared with ground truth. n =61, mean±std. **h,** PSNR of results obtained by M40×, M63×, M19.4×, and M10×, compared with ground truth. The center line represents the median, the box limits represent the lower and upper quartiles, and the whiskers represent 1.5-fold the interquartile range, n =61, mean±std. **i,** Bar chart of LPIPS results at M40×, M63×, M19.4×, and M10× reconstructed from imaging source2, compared with ground truth. n =61, mean±std. Scale bar: 50 μm (M40×, M63×, M19.4×), 200 μm (M10×).


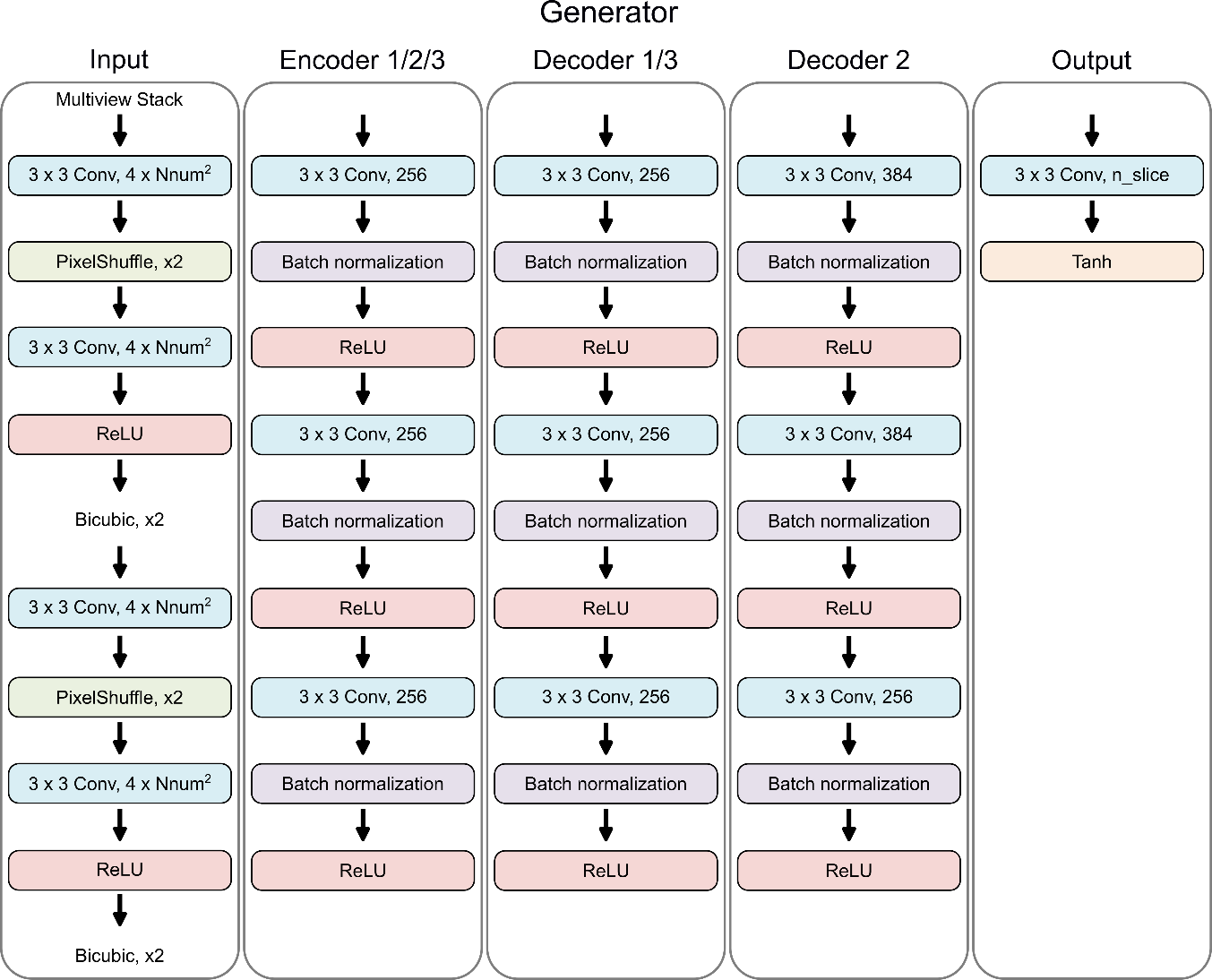


**Fig. S18 | Detailed architecture of the RTU-Net generator.**


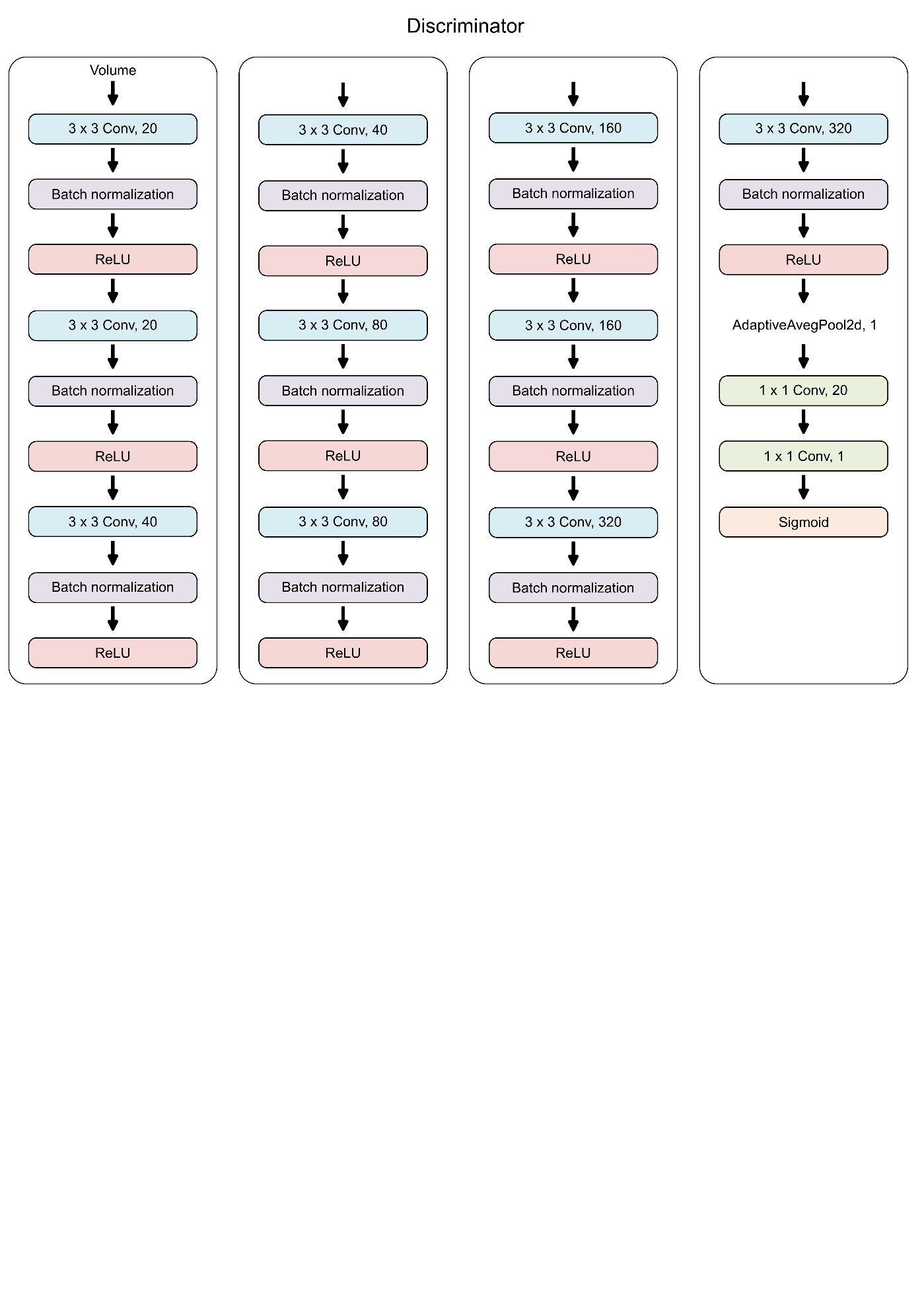


**Fig. S19 | Detailed architecture of the RTU-Net discriminator.**


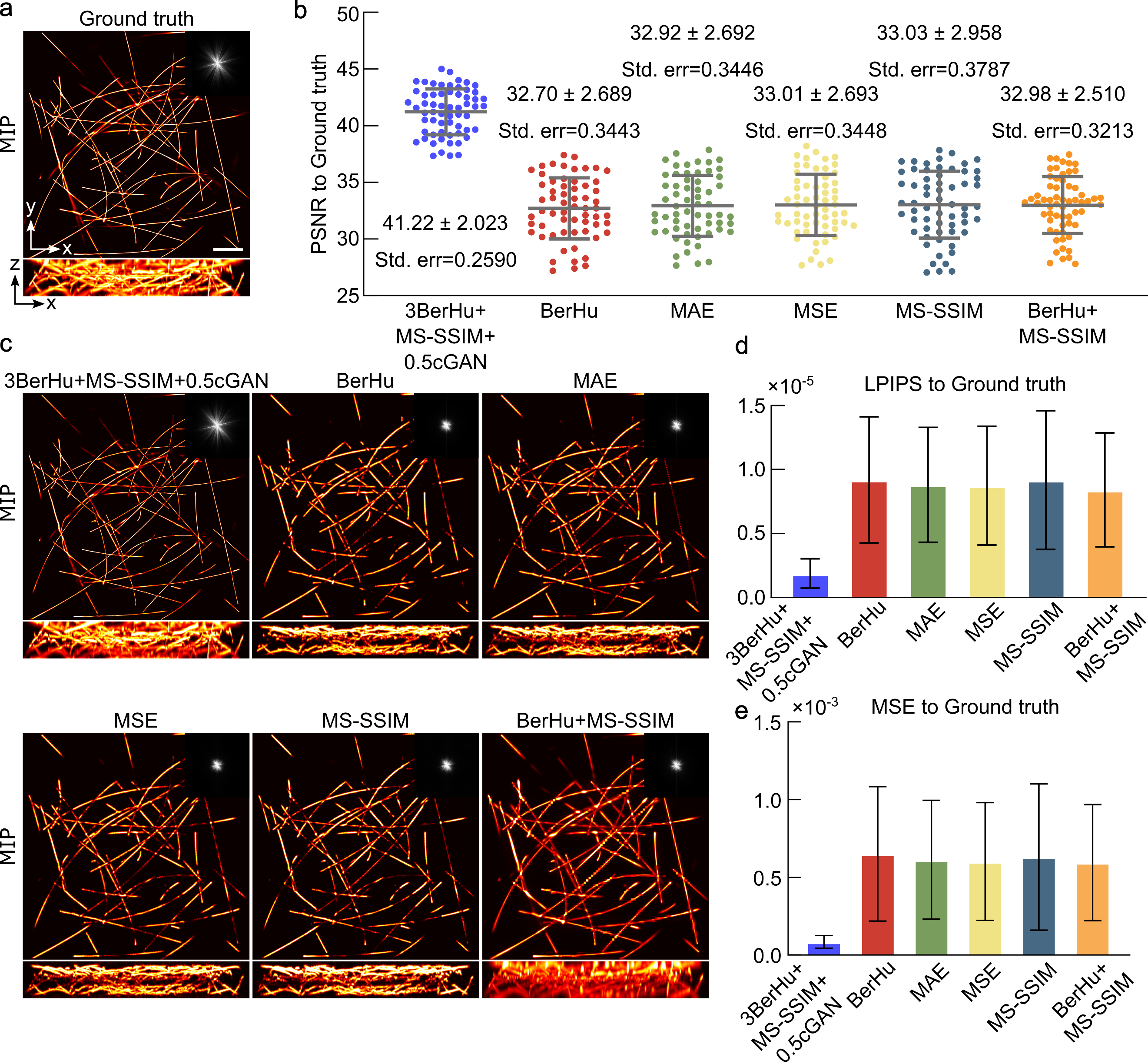


**Fig.** **S20 | Comparison results of RTU-Net on 1 μm tubulins when trained with different loss functions a,** Orthogonal MIPs of 1-μm-diameter synthetic tubulins, acquired by sLFM with a ×63/1.4 NA oil-immersion objective in ideal imaging conditions, regarded as ground truth. **b,** PSNR of results obtained by 3BerHu+MS-SSIM+0.5cGAN, BerHu, MAE, MSE, MS-SSIM, and BerHu+MS-SSIM, compared with ground truth. The center line represents the median, the box limits represent the lower and upper quartiles, and the whiskers represent 1.5-fold the interquartile range, n =61, mean±std. **c,** MIPs obtained by 3BerHu+MS-SSIM+0.5cGAN, BerHu, MAE, MSE, MS-SSIM, and BerHu+MS-SSIM trained on the same type of sample. **d,** LPIPS bar chart from 3BerHu+MS-SSIM+0.5cGAN, BerHu, MAE, MSE, MS-SSIM, and BerHu+MS-SSIM, compared with ground truth. n =61, mean±std. **e,** MSE bar chart from 3BerHu+MS-SSIM+0.5cGAN, BerHu, MAE, MSE, MS-SSIM, and BerHu+MS-SSIM, compared with ground truth. n =61, mean±std. Scale bar: 10 μm.

**
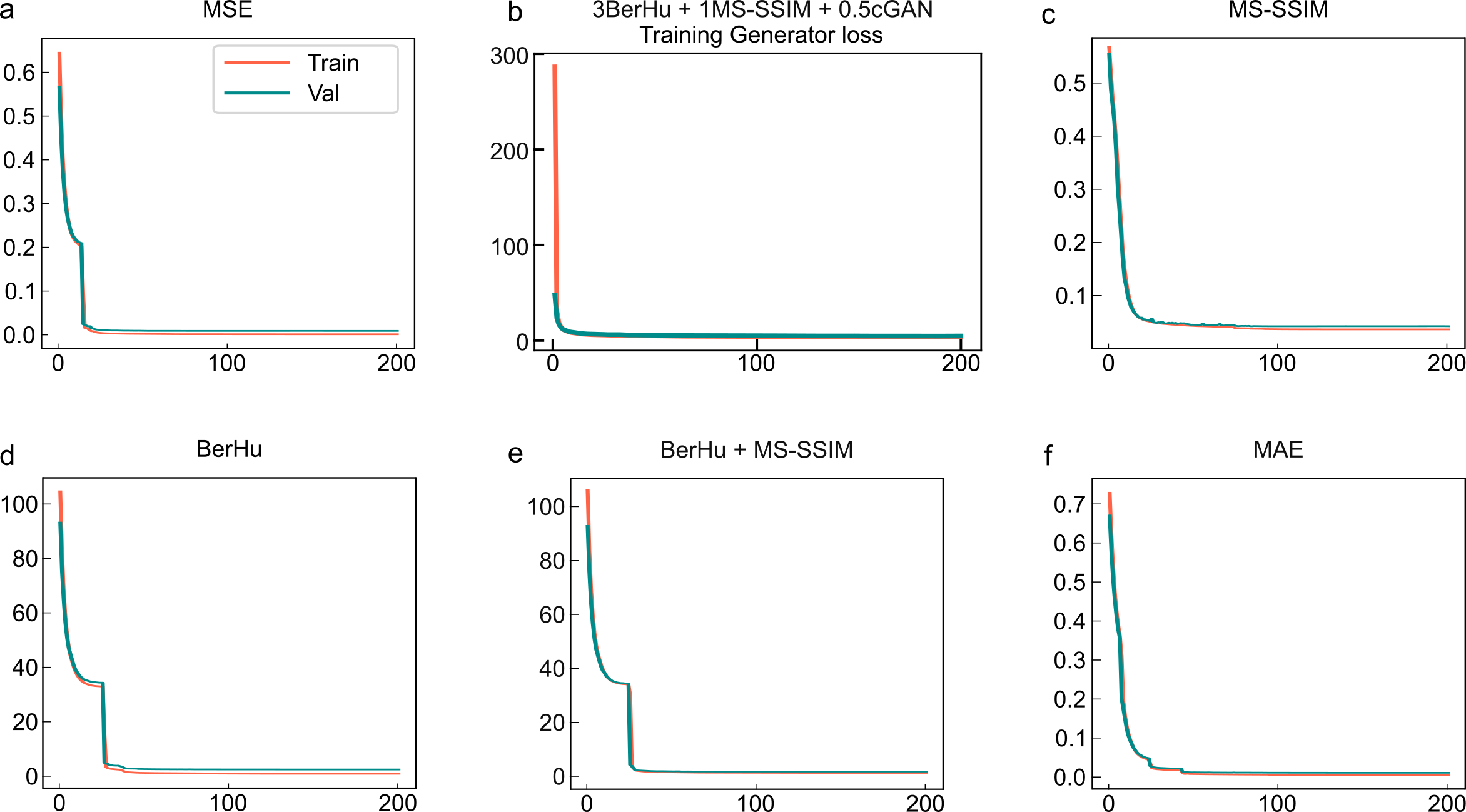
**

**Fig. S21 | Loss changing curves of different loss functions during training process. a,** Loss function of MSE. **b,** Loss function of 3BerHu+1MS-SSIM+0.5cGAN. The resulting generator loss. **c,** Loss function of MS-SSIM. **d,** Loss function of BerHu. **e,** Loss function of BerHu+MS-SSIM. **f,** Loss function of MAE.

**
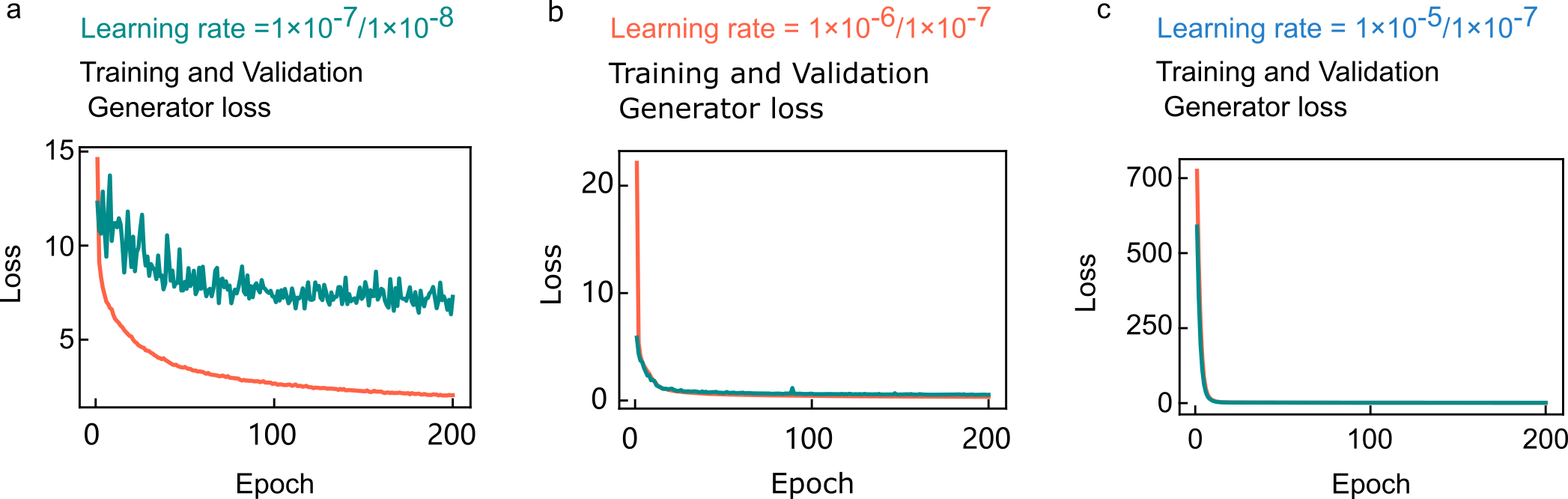
**

**Fig. S2****2 | Loss changing curves of different learning rates during training process.** a, Generator/discriminator Learning rate of 1×10^-7^/1×10^-8^, The resulting generator loss. b, Generator/discriminator Learning rate of 1×10^-6^/1×10^-7^, The resulting generator loss. c, Generator/discriminator Learning rate of 1×10^-5^/1×10^-6^, The resulting generator loss.

**Supplementary Note 1: RTU-Net implementation details**

The RTU-Net is designed to reconstruct 3D volumes directly from 2D light-field raw images. It includes 2 stages: 1. RTU-Net training based on high-resolution 3D images and their synthetic light-fields; 2. Inference on experimental light-field images. Training is required only once for all the following inferences, or it can be skipped if a well-trained RTU-Net has been previously established.

**Table S1. Publicly available data set sources.**

|  | Tubulins (1 μm) | Tubulins (10 μm) | 100-nm beads | Mitochondria |
| --- | --- | --- | --- | --- |
| Source | Lu, Z. Bio-LFSR. Zenodo. <https://doi.org/10.5281/zenodo.7233421>  (2022). | Wang, Z.. VCD-LFM dataset (Version v0). Zenodo. <https://doi.org/10.5281/zenodo.4390067>  (2020) | Lu, Z. Bio-LFSR. Zenodo. <https://doi.org/10.5281/zenodo.7233421>  (2022). | Lu, Z. Bio-LFSR. Zenodo. <https://doi.org/10.5281/zenodo.7233421>  (2022). |
| Experimentation | √ | √ | √ | √ |
| Simulation | × | × | × | × |

**Table S2. Test results of different methods on public datasets.**

|  | RTU-Net | VsLFM | HyLFM-Net | VCD-Net | LFD |
| --- | --- | --- | --- | --- | --- |
| PSNR (1 μm tubulins) Fig.2 | 41.22±2.023 dB | 31.94±1.518 dB | 38.81±1.945 dB | 37.49±1.830 dB | 36.09±1.495 dB |
| LPIPS (1 μm tubulins) Fig.2 | 1.882×10^-6^±1.147×10^-6^ | 1.187×10^-5^±3.765×10^-6^ | 2.414×10^-6^±1.365×10^-6^ | 4.007×10^-6^±2.151×10^-6^ | 4.887×10^-6^±2.530×10^-6^ |
| MSE (1 μm tubulins) Fig.2 | 8.413×10^-5^±4.078×10^-5^ | 6.781×10^-4^±2.247×10^-4^ | 1.156×10^-4^±5.608×10^-5^ | 1.944×10^-4^±8.244×10^-4^ | 2.608×10^-4^±9.341×10^-5^ |
| PSNR (1 μm tubulins) Sup. Fig.4 | 45.22±3.406 dB | 39.45±2.883 dB | 38.57±2.527 dB | 36.58±2.391 dB | 31.30±3.146 dB |
| SSIM (1 μm tubulins) Sup. Fig.4 | 0.9945±0.0054 | 0.9868±0.0084 | 0.9777±0.0157 | 0.9737±0.0163 | 0.9167±0.0277 |
| Pearson correlation (1 μm tubulins) Sup. Fig.4 | 0.9451±0.0279 | 0.7354±0.1089 | 0.8160±0.0557 | 0.5718±0.1236 | 0.6136±0.1034 |
| PSNR (10μm tubulins) | 38.74±1.843 dB | NA | 32.70±1.372 dB | 31.32±1.695 dB | 28.88±1.063 dB |
| SSIM (10μm tubulins) | 0.9922±0.0026 | NA | 0.9427±0.0109 | 0.9119±0.0100 | 0.6663±0.0397 |
| Pearson correlation (10 μm tubulins) | 0.9621±0.0169 | NA | 0.9424±0.0269 | 0.7712±0.05617 | 0.6691±0.0943 |
| SSIM (mitochondria) | 0.8119 | 0.6854 | 0.7362 | 0.7419 | 0.5816 |
| FWHMs (100 nm bead) | 279 nm | 266 nm | 314 nm | 284 nm | 443 nm |
| FWHMs (100 nm beads) | 341.7±60.65 nm | 357.7±70.45 nm | 429.8±121.00 nm | 380.4±95.23 nm | 559.1±271.8 nm |

**Table S3.** **Performance comparison of existing methods.**

|  | VCD-Net | HyLFM-Net | VsLFM | LFD | RTU-Net |
| --- | --- | --- | --- | --- | --- |
| Resolution improvement rate | 2× | 2× | 4× | 1× | 4× |
| PSNR (1 μm tubulins) | 37.49 ± 1.830 dB | 38.81 ± 1.945 dB | 31.94 ±1.518 dB | 36.09 ± 1.495dB | 41.22 ± 2.023 dB |
| LPIPS (1 μm tubulins) | 4.007×10^-6^ ±2.151×10^-6^ | 2.414×10^-6^ ±1.365×10^-6^ | 1.187×10^-5^ ±3.765×10^-6^ | 4.887×10^-6^ ±2.530×10^-6^ | 1.882×10^-6^ ±1.147×10^-6^ |
| MSE (1 μm tubulins) | 1.944×10^-4^ ±8.244×10^-4^ | 1.156×10^-4^ ±5.608×10^-5^ | 6.781×10^-4^ ±2.247×10^-4^ | 2.608×10^-4^ ±9.341×10^-5^ | 8.413×10^-5^ ±4.078×10^-5^ |
| Reconstruction speed | ~ 0.002 s | ~ 0.001 s | ~ 1600 s | ~ 1500 s | ~ 0.002 s |
| # Para | 76.5 M | 35.7 M | 63.6 M | NA | 67.1 M |
| Network structure | Pixel Shuffle  and U-Net | 2D residual blocks and 3D residual blocks | Convolutional layers, Leakey ReLU and Pixel Shuffle | NA | Odd channels skip connections |
| Adaptive loss | NA | NA | NA | NA | √ |
| Multi-scale reconstruction | NA | NA | NA | NA | √ |
| Reconstruction with different imaging sources | NA | NA | NA | NA | √ |
| Ability for multi-scale imaging | NA | NA | NA | NA | √ |

**Table S4. Reconstruction resource consumption vs. Physics-based approach.**

|  | Processing Unit | Implementation Framework | Recon  Memory-Usage | # Para. | Time-consuming(s) |
| --- | --- | --- | --- | --- | --- |
| LFD | GPU | Matlab | 3436 MiB | — | 1495.4551 |
| VsLFM | GPU | Malab/  Pytorch | 8220MiB/  14047MiB | —/63.6M | 1525.1169/  11.2996 |
| RTU-Net | GPU | Pytorch | 30184 MiB | 67.1M | **0.003916** |

**Table S5. Hardware parameters.**

| Name | | Information |
| --- | --- | --- |
| CPU | Type | Intel(R) Xeon(R) Platinum 8269CY CPU @ 2.50GHz |
|  | Core | 26 |
| Disk | Capacity | 6.0T |
| Random Access Memory | Memory | 1006G |
| GPU | Type | NVIDIA A100 PCIe |
|  | Number | 1 |
|  | GPU Memory | 40GB |

**Table S6. 5-round validation results of RTU-Net.**

|  | PSNR↑ | SSIM↑ | LPIPS↓ | MSE↓ | Pearson  Correlation↑ |
| --- | --- | --- | --- | --- | --- |
| R1 | 39.4744 | 0.9738 | 2.508×10^-6^ | 1.307×10^-4^ | 0.8801 |
| R2 | 39.0347 | 0.9742 | 2.586×10^-6^ | 1.326×10^-4^ | 0.8629 |
| R3 | 40.4637 | 0.9802 | 2.070×10^-6^ | 9.683×10^-5^ | 0.9012 |
| R4 | 40.1294 | 0.9772 | 2.238×10^-6^ | 1.082×10^-4^ | 0.8919 |
| R5 | 39.6917 | 0.9766 | 2.507×10^-6^ | 1.151×10^-4^ | 0.8844 |

**Table S7. Detailed microscale optical imaging parameters.**

**（a）63×**

| Parameters |  |
| --- | --- |
| Stack Depth | 61 |
| Axial Oberlap | 0.5 |
| Axial Sampling | 1 |
| dx | 19.38 |
| Nnum | 13 |
| Brightness Adjust | 1 |
| M | 63× |
| NA | 1.4 |
| fml[μm] | 2100 |
| ML Pitch[μm] | 100 |
| n | 1.515 |
| Wavelength | 488 |
| OSR | 3 |
| z-spacing[μm] | 0.2 |
| z-min[μm] | -6 |
| z-max[μm] | 6 |

**（b）40×**

| Parameters |  |
| --- | --- |
| Stack Depth | 61 |
| Axial Oberlap | 0.5 |
| Axial Sampling | 1 |
| dx | 15.23 |
| Nnum | 11 |
| Brightness Adjust | 1 |
| M | 40× |
| NA | 0.8 |
| fml[μm] | 3500 |
| ML Pitch[μm] | 150 |
| n | 1.33 |
| Wavelength | 532 |
| OSR | 3 |
| z-spacing[μm] | 1 |
| z-min[μm] | -30 |
| z-max[μm] | 30 |

**Table S8. Detailed microscale optical imaging parameters.**

| Parameters |  |
| --- | --- |
| Stack Depth | 31 |
| Axial Oberlap | 0.5 |
| Axial Sampling | 1 |
| dx | 15.23 |
| Nnum | 15 |
| Brightness Adjust | 1 |
| M | 19.4× |
| NA | 1.0 |
| fml[μm] | 1000 |
| ML Pitch[μm] | 100 |
| n | 1.33 |
| Wavelength | 532 |
| OSR | 3 |
| z-spacing[μm] | 6.5 |
| z-min[μm] | -65 |
| z-max[μm] | 65 |

**Table S9. Detailed mesoscale optical imaging parameters**

| Parameters |  |
| --- | --- |
| Stack Depth | 71 |
| Axial Oberlap | 0.5 |
| Axial Sampling | 1 |
| dx | 15.23 |
| Nnum | 15 |
| Brightness Adjust | 1 |
| M | 10× |
| NA | 0.4 |
| fml[μm] | 1300 |
| ML Pitch[μm] | 100 |
| n | 1.33 |
| Wavelength | 520 |
| OSR | 3 |
| z-spacing[μm] | 6 |
| z-min[μm] | -210 |
| z-max[μm] | 210 |

**Table S10. Detailed macroscale optical imaging parameters**

| Parameters |  |
| --- | --- |
| Stack Depth | 125 |
| Axial Oberlap | 0.5 |
| Axial Sampling | 1 |
| dx | 19.38 |
| Nnum | 19 |
| Brightness Adjust | 1 |
| M | 0.5× |
| NA | 0.045 |
| fml[μm] | 500 |
| ML Pitch[μm] | 100 |
| n | 1.33 |
| Wavelength | 532 |
| OSR | 3 |
| z-spacing[μm] | 200 |
| z-min[μm] | -12400 |
| z-max[μm] | 12400 |

**Table S11. Ablation test with different skip concatenations.**

| Type | PSNR↑ | SSIM↑ | LPIPS↓ | # Para. | *T*_Train_ (h) | *T*_Eval_ (s) |
| --- | --- | --- | --- | --- | --- | --- |
| Conventional concatenation | 37.53 ± 1.600 | 0.9660 ±0.00856 | 3.682**×**10^-6^ **±** .645**×**10^-6^ | 76.8M | 16.7 | 0.002241 |
| Odd channels skip connections | 38.62 ± 1.429 | 0.9729 ± 0.00886 | 2.836**×**10^-6^ **±** 1.235**×**10^-6^ | 67.1M | 16 | 0.001931 |
| Even channels skip connections | **41.22 ± 2.023** | **0.9818 ± 0.006121** | **1.882×10^-6^ ± 1.147×10^-6^** | 67.1M | 16 | **0.001916** |

**Table S12. Detailed Architecture of the RTU-Net-Generator.**

| **Alias** | | **RTU-Net-Generator** |
| --- | --- | --- |
| E1 |  | Conv n256f3s1 |
|  |  | Batch normalization |
|  | layer1 | ReLU |
|  |  | Conv n256f3s1 |
|  |  | Batch normalization |
|  | layer2 | ReLU |
|  |  | Conv n256f3s1 |
|  |  | Batch normalization |
|  | layer3 | ReLU |
| E2 |  | Conv n256f3s1 |
|  |  | Batch normalization |
|  | layer4 | ReLU |
|  |  | Conv n256f3s1 |
|  |  | Batch normalization |
|  | layer5 | ReLU |
|  |  | Conv n256f3s1 |
|  |  | Batch normalization |
|  | layer6 | ReLU |
| E3 |  | Conv n256f3s1 |
|  |  | Batch normalization |
|  | layer7 | ReLU |
|  |  | Conv n256f3s1 |
|  |  | Batch normalization |
|  | layer8 | ReLU |
|  |  | Conv n256f3s1 |
|  |  | Batch normalization |
|  | layer9 | ReLU |
| bottleneck |  | Conv n256f3s1 |
|  |  | Batch normalization |
|  | layer10 | ReLU |
|  |  | Conv n256f3s1 |
|  |  | Batch normalization |
|  | layer11 | ReLU |
|  |  | Conv n256f3s1 |
|  |  | Batch normalization |
|  | layer12 | ReLU |
| D1 |  | Conv n256f3s1 |
|  |  | Batch normalization |
|  | layer13 | ReLU |
|  |  | Conv n256f3s1 |
|  |  | Batch normalization |
|  | layer14 | ReLU |
|  |  | Conv n256f3s1 |
|  |  | Batch normalization |
|  | layer15 | ReLU |
| D2 |  | Concat [ layer9[::2], layer15 ] |
|  |  | Conv n384f3s1 |
|  |  | Batch normalization |
|  | layer16 | ReLU |
|  |  | Conv n384f3s1 |
|  |  | Batch normalization |
|  | layer17 | ReLU |
|  |  | Conv n256f3s1 |
|  |  | Batch normalization |
|  | layer18 | ReLU |
| D3 |  | Conv n256f3s1 |
|  |  | Batch normalization |
|  | layer19 | ReLU |
|  |  | Conv n256f3s1 |
|  |  | Batch normalization |
|  | layer20 | ReLU |
|  |  | Conv n256f3s1 |
|  |  | Batch normalization |
|  | layer21 | ReLU |
| Out |  | Concat [ layer3[::2], layer21 ] |
|  |  | Conv nn_slicef3s1 |
|  | layer22 | Tanh |

“Conv” is the abbreviation for convolutional 2D layer, the parameters of which are number of output channels (n), filter size (f), and stride (s). “Concat” is abbreviation for concatenation layer, which combines the feature maps of the two input layers along the “channel” dimension,[::2] represents the even “channel” dimension of the layer.

**Table S13. Detailed Architecture of the RTU-Net-Discriminator.**

| Alias | | RTU-Net-Discriminator |
| --- | --- | --- |
| DC1 |  | Conv n20f3s1 |
|  |  | Batch normalization |
|  | layer1 | ReLU |
|  |  | Conv n20f3s1 |
|  |  | Batch normalization |
|  | layer2 | ReLU |
| DC2 |  | Conv n40f3s1 |
|  |  | Batch normalization |
|  | layer3 | ReLU |
|  |  | Conv n40f3s1 |
|  |  | Batch normalization |
|  | layer4 | ReLU |
| DC3 |  | Conv n80f3s1 |
|  |  | Batch normalization |
|  | layer5 | ReLU |
|  |  | Conv n80f3s1 |
|  |  | Batch normalization |
|  | layer6 | ReLU |
| DC4 |  | Conv n160f3s1 |
|  |  | Batch normalization |
|  | layer7 | ReLU |
|  |  | Conv n160f3s1 |
|  |  | Batch normalization |
|  | layer8 | ReLU |
| DC5 |  | Conv n320f3s1 |
|  |  | Batch normalization |
|  | layer9 | ReLU |
|  |  | Conv n320f3s1 |
|  |  | Batch normalization |
|  | layer10 | ReLU |
| Adapt | layer11 | AdaptiveAvegPool2d size1 |
| Dense | layer12 | Conv n20f1s1 |
|  |  | Conv n1f1s1 |
|  | layer13 | Sigmoid |

**Table S14. Ablation test with different loss function weights.**

| Loss function weights | PSNR↑ | SSIM↑ | LPIPS↓ |
| --- | --- | --- | --- |
| $\alpha$=1, $\beta$=1, $\gamma$=0.5 | 33.20 ± 2.189 | 0.9345 ± 0.01560 | 9.231×10^-6^ ± 4.024×10^-6^ |
| $\alpha$=1, $\beta$=2, $\gamma$=0.5 | 32.31 ± 2.746 | 0.9323 ± 0.01554 | 9.577×10^-6^ ± 4.437×10^-6^ |
| $\alpha$=1, $\beta$=3, $\gamma$=0.5 | 31.73 ± 2.851 | 0.9277 ± 0.01651 | 10.00×10^-6^ ± 4.712×10^-6^ |
| $\alpha$=2, $\beta$=1, $\gamma$=0.5 | 32.95 ± 2.204 | 0.9339 ± 0.01556 | 9.134×10^-6^ ± 3.886×10^-6^ |
| $\alpha$=3, $\beta$=1, $\gamma$=1.5 | 32.61 ± 2.688 | 0.9329 ± 0.01647 | 9.854×10^-6^ ± 4.631×10^-6^ |
| $\alpha$=3, $\beta$=1, $\gamma$=1.0 | 33.15 ± 2.430 | 0.9330 ± 0.01654 | 9.882×10^-6^ ± 4.500×10^-6^ |
| $\alpha$=3, $\beta$=1, $\gamma$=0.5 | **41.22 ± 2.023** | **0.9818 ± 0.006121** | **1.882×10^-6^ ± 1.147×10^-6^** |

**Table S15. Hyperparameters used in training process.**

| Models | Learning  rate | Optimizer | Activation  function | Weight  decay | Batch  size | Implementation  Framework | Epoch |
| --- | --- | --- | --- | --- | --- | --- | --- |
| Generator | 1x10^-5^ | AdamW | ReLU,  Tanh | 1x10^-2^ | 2 | Pytorch | 200 |
| Discriminator | 1x10^-6^ | AdamW | ReLU,  Sigmoid | 1x10^-2^ | 2 | Pytorch | 200 |

**Table S16. Ablation test with different loss function weights.**

| Loss function | PSNR↑ | SSIM↑ | LPIPS↓ |
| --- | --- | --- | --- |
| MSE | 33.01 ± 2.693 | 0.9396 ± 0.01730 | 8.744×10^-6^ ± 4.637×10^-6^ |
| MAE | 32.92 ± 2.692 | 0.9402 ± 0.01705 | 8.805×10^-6^ ± 4.485×10^-6^ |
| BerHu | 32.70 ± 2.689 | 0.9373 ± 0.01655 | 9.195×10^-6^ ± 4.923×10^-6^ |
| MS-SSIM | 33.03 ± 2.958 | 0.9424 ± 0.01722 | 9.172×10^-6^ ± 5.416×10^-6^ |
| 3BerHu + MS-SSIM | 32.98 ± 2.510 | 0.9395 ± 0.01644 | 8.412×10^-6^ ± 4.450×10^-6^ |
| 0.5cGAN + 3BerHu + MS-SSIM | **41.22 ± 2.023** | **0.9818 ± 0.00612** | **1.882×10^-6^ ± 1.147×10^-6^** |

**Table S17. Ablation test with different dataset sizes.**

| Data size | PSNR↑ | SSIM↑ | LPIPS↓ | MSE↓ | Pearson  Correlation↑ |
| --- | --- | --- | --- | --- | --- |
| 2000 | 40.22 | 0.9769 | 1.945×10^-6^ | 8.621×10^-4^ | 0.9355 |
| 4000 | 41.54 | 0.9833 | 1.687×10^-6^ | 7.325×10^-5^ | 0.9460 |
| 6000 | 41.83 | 0.9876 | 1.561×10^-6^ | 6.373×10^-5^ | 0.9476 |

**Table S18. Ablation test with different optimizers.**

| Opt. | PSNR↑ | SSIM↑ | LPIPS↓ | MSE↓ | Peason  Correlation↑ |
| --- | --- | --- | --- | --- | --- |
| Rprop | 36.11 | 0.9526 | 5.626×10^-6^ | 2.649×10^-4^ | 0.6956 |
| SGD | 27.14 | 0.0706 | 1.554×10^-5^ | 1.947×10^-3^ | 0.0339 |
| AdamW | **41.22** | **0.9818** | **1.882×10^-6^** | **8.413×10^-5^** | **0.9429** |

**Table S19. Ablation test with different batch sizes.**

| Batch size | PSNR↑ | SSIM↑ | LPIPS↓ | MSE↓ | Peason  Correlation↑ |
| --- | --- | --- | --- | --- | --- |
| 2 | 41.22 | 0.9818 | 1.882×10^-6^ | 8.413×10^-5^ | **0.9429** |
| 4 | 40.74 | 0.9825 | 1.799×10^-6^ | 9.320×10^-5^ | 0.9160 |
| 8 | 40.70 | **0.9833** | 1.944×10^-6^ | 9.729×10^-5^ | 0.9215 |
| 16 | **41.26** | 0.9766 | **1.642×10^-6^** | **8.373×10^-5^** | 0.9256 |

**Table S20. Ablation test with different learning rates.**

| Learning rate  G/D | PSNR↑ | SSIM↑ | LPIPS↓ | MSE↓ | Peason  Correlation↑ |
| --- | --- | --- | --- | --- | --- |
| 1×10^-5^/1×10^-6^ | 38.73 | 0.9732 | 2.940×10^-6^ | 1.425×10^-4^ | 0.8437 |
| 1×10^-6^/1×10^-7^ | **41.22** | **0.9818** | **1.882×10^-6^** | **8.413×10^-5^** | **0.9429** |
| 1×10^-7^/1×10^-8^ | 34.08 | 0.9379 | 8.359×10^-6^ | 4.181×10^-4^ | 0.3040 |
